# A mechanical signal transmitted by the flagellum controls signalling in *Bacillus subtilis*

**DOI:** 10.1111/mmi.12342

**Published:** 2013-08-14

**Authors:** Lynne S Cairns, Victoria L Marlow, Emma Bissett, Adam Ostrowski, Nicola R Stanley-Wall

**Affiliations:** Division of Molecular Microbiology, College of Life Sciences, University of DundeeDundee, DD1 5EH, UK

## Abstract

In the natural environment bacteria predominantly live adhered to a surface as part of a biofilm. While many of the components needed for biofilm assembly are known, the mechanism by which microbes sense and respond to contact with a surface is poorly understood. *Bacillus subtilis* is a Gram-positive model for biofilm formation. The DegS–DegU two-component system controls several multicellular behaviours in *B. subtilis*, including biofilm formation. Here we identify the *B. subtilis* flagellum as a mechanosensor that activates the DegS–DegU regulatory pathway. Inhibition of flagellar rotation by deletion or mutation of the flagellar stator gene, *motB*, results in an increase in both *degU* transcription and DegU∼P driven processes, namely exoprotease production and poly-γ-dl-glutamic acid biosynthesis. Similarly, inhibition of flagellar rotation by engaging the flagellar clutch or by tethering the flagella with antibodies also promotes an increase in *degU* transcription that is reflective of increased DegU∼P levels in the cell. Collectively, these findings strongly indicate that inhibition of flagellar rotation acts as a mechanical trigger to activate the DegS–DegU two-component signal transduction system. We postulate that inhibition of flagellar rotation could function as a mechanical trigger to activate bacterial signal transduction cascades in many motile bacteria upon contact with a surface.

## Introduction

Persistent adhesion of bacterial cells to a surface is the first step in the formation of a biofilm – a complex community of bacteria encased in a self-produced exopolymeric matrix (Flemming and Wingender, 2010). The settlement of microbes on a surface within the confines of a biofilm can confer many advantages to the population, including increased access to nutrients and protection from environmental stress (Costerton *et al*., 1995). Despite significant recent advances in our understanding of the regulatory pathways and key building blocks required for the nucleation and growth of biofilms for many species of bacteria (Flemming and Wingender, 2010; Lopez *et al*., 2010; Vlamakis *et al*., 2013), it is not fully understood how a motile cell senses and responds to a surface.

The bacterial flagellum is a complex molecular machine comprised of over 30 different proteins and is organized into three main structural domains: the basal body, hook and filament (Chevance and Hughes, 2008). The filament acts as a propeller to drive movement and is powered by a rotary motor that comprises stator and rotor protein complexes and can be driven by proton-motive or sodium-motive force (Manson *et al*., 1977; Chernyak *et al*., 1983). Torque is generated by specific interactions between the rotor and stator components (Zhou *et al*., 1998a). The stator complex, which in *Escherichia coli* comprises a complex of four MotA proteins and two MotB proteins, forms two proton channels (Braun *et al*., 1999; Kojima and Blair, 2004). It is thought that proton flux through the channels triggers a conformational change that alters the electrostatic interactions between MotA and the rotor protein FliG, resulting in torque generation (Zhou *et al*., 1998a). As well as being required as a mechanical device for propulsion, the flagellum is also crucial for biofilm development in many bacterial species due to its role in the initial stages of surface adhesion (O'Toole *et al*., 2000).

*Bacillus subtilis* is a Gram-positive, non-pathogenic, soil-dwelling bacterium that has emerged as a model organism for the study of biofilm formation (Vlamakis *et al*., 2013). The development of the *B. subtilis* biofilm is tightly controlled and requires the activation of three transcriptional regulators: ComA (Lopez *et al*., 2009c), Spo0A (Branda *et al*., 2001; Hamon and Lazazzera, 2001) and DegU (Stanley and Lazazzera, 2005). Previous studies have delineated many of the signals required to activate both ComA and Spo0A. At high cell density the quorum sensing transcription factor, ComA is phosphorylated, resulting in production of the lipopeptide, surfactin (Nakano *et al*., 1991). The production of surfactin, along with other signals that induce potassium leakage (Lopez *et al*., 2009a; Shank and Kolter, 2011) and osmotic stress (Rubinstein *et al*., 2012), triggers activation of a multicomponent phosphorelay which begins with the phosphorylation of one or more sensor histidine kinases (namely KinA to KinE, each of which has a unique role in signal perception) (see Vlamakis *et al*., 2013) and culminates in phosphorylation of Spo0A (Burbulys *et al*., 1991). The level of phosphorylated Spo0A (hereafter Spo0A∼P) within the cell dictates which bacterial behaviour will be stimulated or repressed (Fujita *et al*., 2005; Lopez *et al*., 2009b). Biofilm formation by *B. subtilis* requires a low level of Spo0A∼P to indirectly promote the transcription of the *tapA-sipW-tasA* and *epsA-O* operons (Fujita *et al*., 2005; Chai *et al*., 2011), which encode the extracellular biofilm matrix amyloid protein, TasA and proteins required for the synthesis of the biofilm matrix exopolysaccharide (EPS) respectively (Vlamakis *et al*., 2013). The third component required for *B. subtilis* biofilm formation is the hydrophobic coat protein, BslA (formerly YuaB) (Kobayashi and Iwano, 2012; Hobley *et al*., 2013). Transcription of the *bslA* gene is indirectly activated by phosphorylated DegU (hereafter DegU∼P) (Kobayashi, 2007; Ostrowski *et al*., 2011).

DegU is a response regulator that is phosphorylated by its cytoplasmic cognate sensor histidine kinase, DegS (Dahl *et al*., 1991). DegU∼P is a pleiotropic regulator that controls a myriad of processes, including flagella-based motility (Amati *et al*., 2004; Verhamme *et al*., 2007; Hsueh *et al*., 2011; Patrick and Kearns, 2012), biofilm formation (Kobayashi, 2007; Verhamme *et al*., 2007), exoprotease production (Dahl *et al*., 1992) and biosynthesis of the exopolymer poly-γ-dl-glutamic acid (hereafter γ-PGA) (Stanley and Lazazzera, 2005). The ability of DegU∼P to promote both motility and γ-PGA production is dependent on the small protein SwrA (Kearns *et al*., 2004; Stanley and Lazazzera, 2005; Calvio *et al*., 2008; Osera *et al*., 2009). The main role of SwrA is to regulate the number of flagellar hook-basal bodies in the cell (Kearns and Losick, 2005; Guttenplan *et al*., 2013). It is thought that the ability of DegU∼P to regulate several different processes is underpinned by variation in promoter affinities (Kobayashi, 2007; Murray *et al*., 2009). A small protein, DegQ, aids the transfer of the phosphoryl moiety from DegS to DegU (Kobayashi, 2007) with recent work suggesting that this is due to the ability of DegQ to stabilize the phosphorylated form of DegS in the presence of DegU (Do *et al*., 2011). Transcription of *degQ* is positively regulated by ComA and thus increases in response to cell density, thereby ensuring that DegU∼P levels also rise as growth approaches stationary phase (Msadek *et al*., 1991).

While many aspects of DegU activation and regulation are understood (for a review see Murray *et al*., 2009), the signal sensed by DegS to trigger phosphorylation of DegU has remained somewhat elusive. Previous studies have identified a link between the activity of the DegS–DegU system and osmolarity (Ruzal and Sanchez-Rivas, 1998), the structural maintenance of the chromosome (SMC)–ScpA–ScpB complex (Dervyn *et al*., 2004), ClpCP mediated proteolytic degradation (Ogura and Tsukahara, 2010), the RapG-PhrG quorum sensing system (Ogura *et al*., 2003) and, more recently, the completion status of the flagellar basal body (Hsueh *et al*., 2011). The aim of this work was to investigate the link between flagellar assembly and phosphorylation of DegU. We hypothesized that inhibition of flagellar rotation might trigger activation of the DegS–DegU two-component system. This would provide a mechanism to allow motile cells to detect and respond to the presence of a surface during the initial stages of biofilm formation. The data presented here identifies the *B. subtilis* flagellum as a mechanosensor. Deletion of the flagellar stator gene, *motB*, triggered an increase in DegU∼P levels, exemplified by an upregulation of *degU* transcription and two distinct DegU∼P driven processes, namely exoprotease production and γ-PGA biosynthesis. Further experiments designed to perturb flagellar rotation by genetic and non-genetic methods also resulted in elevated DegU∼P levels within the cell. We conclude that the DegS–DegU two-component regulatory system is activated by the lack of flagellar rotation. As the flagellar structure is highly conserved between microbial species, the arrest of flagellar rotation may present a mechanism by which many flagellated organisms detect and respond to a surface.

## Results

### Deletion of *motB* is associated with increased γ-PGA biosynthesis

**Figure 1:**
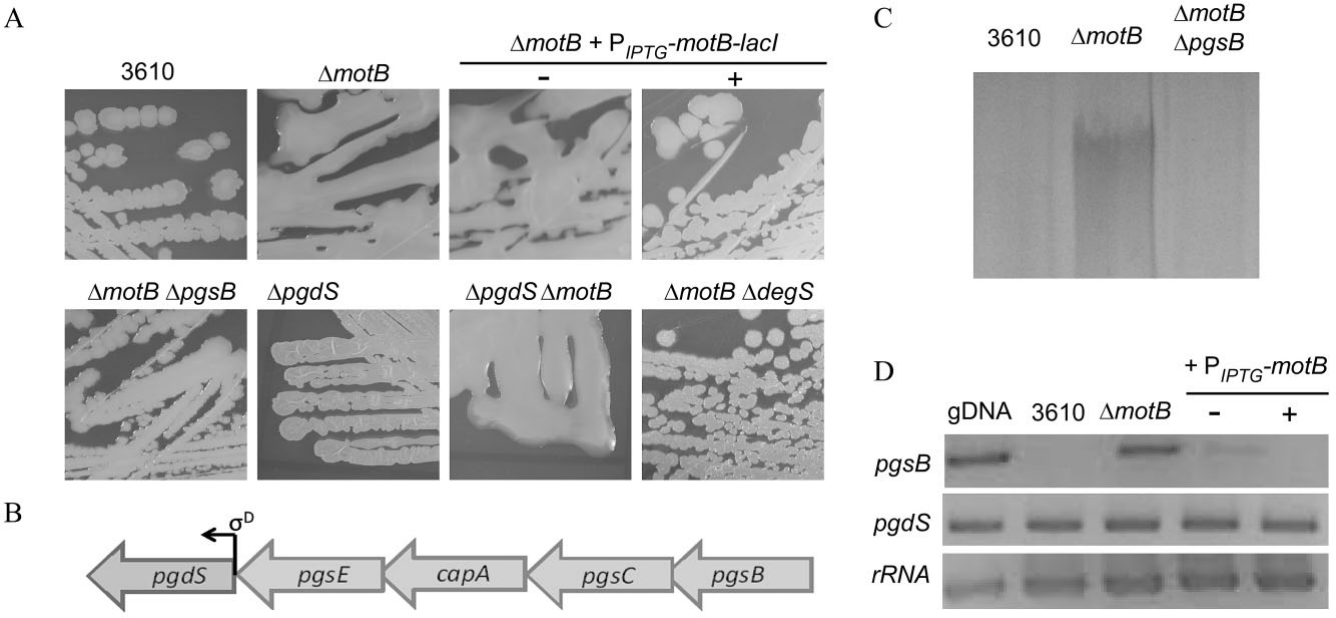
Deletion of *motB* from the chromosome is associated with γ-PGA production.A. Colony morphology of 3610 (wild-type), Δ*motB* (NRS3494), Δ*motB* + *amyE::*P_*IPTG*_-*motB*-*lacI* (NRS3775) grown on LB agar plate in the absence or presence of 50 μM IPTG, Δ*motB* + *pgsB::spc* (NRS3434), Δ*pgdS* (NRS3347), Δ*pgdS*Δ*motB* (NRS3348) and Δ*motB* + *degS::cml* (NRS3398).B. Schematic diagram of the γ-PGA synthesis operon and γ-PGA hydrolase gene. Arrows represent open reading frames (ORF), with the direction of the arrow indicating the direction of the ORF. The bent arrow represents the promoter located before the *pgdS* gene, which is driven by the alternative sigma factor, σ^D^, as indicated.C. SDS-PAGE of γ-PGA collected from cultures of NCIB3610, Δ*motB* (NRS3494) and Δ*motB* + *pgsB::spc* (NRS3434) grown to the onset of stationary phase.D. Reverse-transcription-PCR analysis of *pgsB* and *pgdS*. Regions of DNA internal to *pgsB* and *pgdS* were amplified from cDNA generated from the wild-type (NCIB3610), Δ*motB* (NRS3494) and Δ*motB* + *amyE::*P_*IPTG*_-*motB*-*lacI* (NRS3775) grown in the absence and presence of 50 μM IPTG. Genomic DNA (gDNA) was used as a positive control for the PCR reaction and the ribosomal 16S rRNA was amplified as an internal control.

To test if flagellar rotation was linked to the activity of the DegS–DegU two-component system, an in-frame non-polar deletion in the flagellar stator gene, *motB* was constructed (NRS3494). Disruption of the flagellar stator genes perturbs motility but has no effect on biosynthesis of the flagellum itself (Chevance and Hughes, 2008). Consistent with this, the Δ*motB* strain synthesized flagella but displayed a non-motile phenotype (Fig. S1A and C). The observed motility defect was complemented upon re-introduction of the *motB* coding sequence on the chromosome under the control of an IPTG-inducible promoter (Phy-spank) at the non-essential *amyE* locus (NRS3775) verifying the specificity in the deletion (Fig. S1B). Strikingly, as shown in Fig. 1A, the Δ*motB* strain displayed a mucoid colony phenotype on LB plates after growth overnight. The mucoid colony morphology was specific to deletion of *motB* as the colony morphology reverted to the flat dry phenotype exhibited by the wild-type strain upon heterologous expression of *motB* (Fig. 1A). Production of the exopolymer γ-PGA has been linked with mucoid colony morphology in *B. subtilis* (Stanley and Lazazzera, 2005). The relationship between the mucoid colony morphology of the *motB* deletion strain and γ-PGA production was confirmed as γ-PGA could be biochemically extracted from the culture supernatant collected at the onset of stationary phase upon deletion of *motB* (Fig. 1C and Fig. S1D).

Increased γ-PGA biosynthesis in the absence of *motB* was predicted to be the consequence of: (i) decreased hydrolysis of γ-PGA and/or (ii) increased biosynthesis of γ-PGA. γ-PGA biosynthesis is driven by the protein products of the *pgsB* operon, while turnover is catalysed by the endo-γ-glutamyl peptidase, PgdS (Fig. 1B) (Candela and Fouet, 2006). Consistent with deletion of *motB* triggering increased γ-PGA biosynthesis, reverse-transcription (RT)-PCR analysis showed that transcription of the *pgsB* coding region could not be detected in NCIB3610, but was detectable upon deletion of *motB* (Fig. 1D). Furthermore, the mucoid phenotype of the Δ*motB* strain was abolished upon disruption of *pgsB* (Fig. 1A). In contrast, the *pgdS* coding region was transcribed in NCIB3610, Δ*motB* and the complemented strain (Fig. 1D). Consistent with these data, deletion of *pgdS* did not result in a mucoid colony morphology on LB agar plates indicating that the *pgdS* deletion strain did not phenocopy the *motB* strain (Fig. 1A). Collectively, these data demonstrate that deletion of *motB* triggers increased biosynthesis of the exopolymer γ-PGA.

### Biosynthesis of γ-PGA is triggered by high levels of DegU∼P in *B. subtilis* NCIB3610

**Figure 2:**
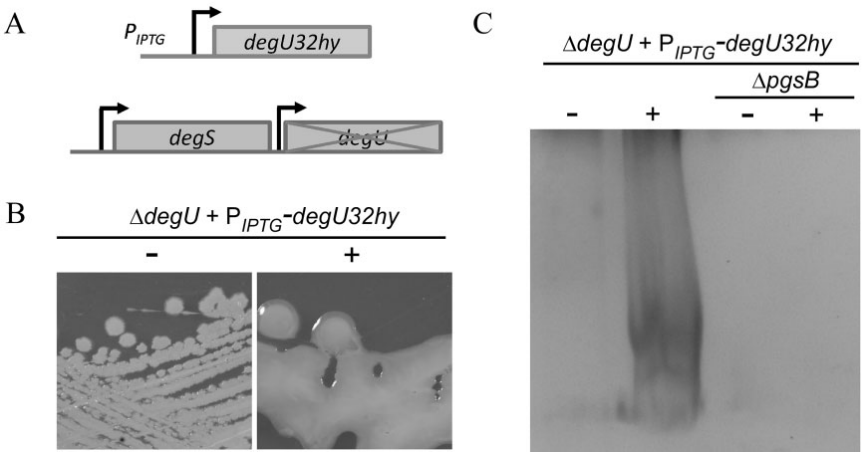
Increasing DegU∼P levels allows γ-PGA production in NCIB3610.A. Schematic diagram of the construction of the Δ*degU* + *amyE::*P_*IPTG*_-*degU32hy*-*lacI* strain (NRS1325). Bent arrows represent the promoters located before each gene.B. Colony morphology of the Δ*degU* + *amyE::*P_*IPTG*_-*degU32hy*-*lacI* (NRS1325) strain without and with induction with 25 μM IPTG.C. SDS-PAGE of γ-PGA collected from cultures of Δ*degU* + *amyE::*P_*IPTG*_-*degU32hy*-*lacI* (NRS1325) and Δ*degU* + *amyE::*P_*IPTG*_-*degU32hy*-*lacI* + *pgsB::spc* (NRS3770) grown to the onset of stationary phase in the absence or presence of 25 μM IPTG.

Consistent with our hypothesis that flagellar rotation might control the activity of the DegS–DegU two-component system, production of γ-PGA is linked with high levels of DegU∼P (Stanley and Lazazzera, 2005; Osera *et al*., 2009). Transcription of the γ-PGA synthetase gene, *pgsB* is directly regulated by DegU∼P (Ohsawa *et al*., 2009), supporting the hypothesis that γ-PGA biosynthesis is increased in the Δ*motB* strain due to an increase in the level of DegU∼P. It is worth noting that in certain *B. subtilis* isolates, such as R0-FF-1, γ-PGA is a component of the biofilm matrix (Stanley and Lazazzera, 2005; Morikawa *et al*., 2006). However, in NCIB3610 this is not the case as γ-PGA is not synthesized, despite the presence of an intact γ-PGA biosynthetic operon on the chromosome (Branda *et al*., 2006; Srivatsan *et al*., 2008; Earl *et al*., 2012). We therefore hypothesized that in NCIB3610 the DegU∼P levels are suppressed and that this suppression was alleviated upon deletion of *motB*. To test if directly increasing the level of DegU∼P was sufficient to allow biosynthesis of γ-PGA by NCIB3610, we used a synthetic strain of NCIB3610 that contained a disruption in the native *degU* gene and carried an allele of *degU* containing an H^12^L amino acid mutation (*degU32hy*) under the control of an IPTG-inducible promoter at the non-essential *amyE* locus (NRS1325) (Verhamme *et al*., 2007) (Fig. 2A). The *degU32 hy* gene encodes a DegU variant that is described as exhibiting a slower rate of dephosphorylation by comparison with the wild-type protein, thus increasing the level of DegU∼P in the cell (Dahl *et al*., 1992). As shown in Fig. 2B, induction of *degU32hy* expression with 25 μM IPTG resulted in a highly mucoid colony phenotype. The relationship between the mucoid colony morphology and γ-PGA production was confirmed as γ-PGA could be biochemically extracted from culture supernatant collected at the onset of stationary phase upon induction of *degU32 hy* expression (Fig. 2C). Moreover, disruption of the γ-PGA synthetase gene, *pgsB*, in this background abolished γ-PGA production as assessed by the presence of flat dry colonies (data not shown) and a lack of exopolymer extraction from the culture supernatant (Fig. 2C). Intriguingly, a strain carrying the *degU32 hy* allele retained γ-PGA biosynthesis in the absence of *degS* as defined by a mucoid colony morphology (data not shown). These findings indicate that DegU H^12^L can be phosphorylated by another kinase or by acetyl phosphate (Wolfe *et al*., 2003; 2008) as has been suggested previously for the accumulation of low levels of native DegU∼P in the absence of *degS* (Kobayashi, 2007; Verhamme *et al*., 2007). These data highlight that increasing the level of DegU∼P in NCIB3610 results in γ-PGA production. This is replicated upon deletion of *motB*, suggesting that upon inhibition of flagellar rotation DegU∼P levels are increased.

### The Δ*motB* strain shows increased *degU* transcription and protease production

**Figure 3:**
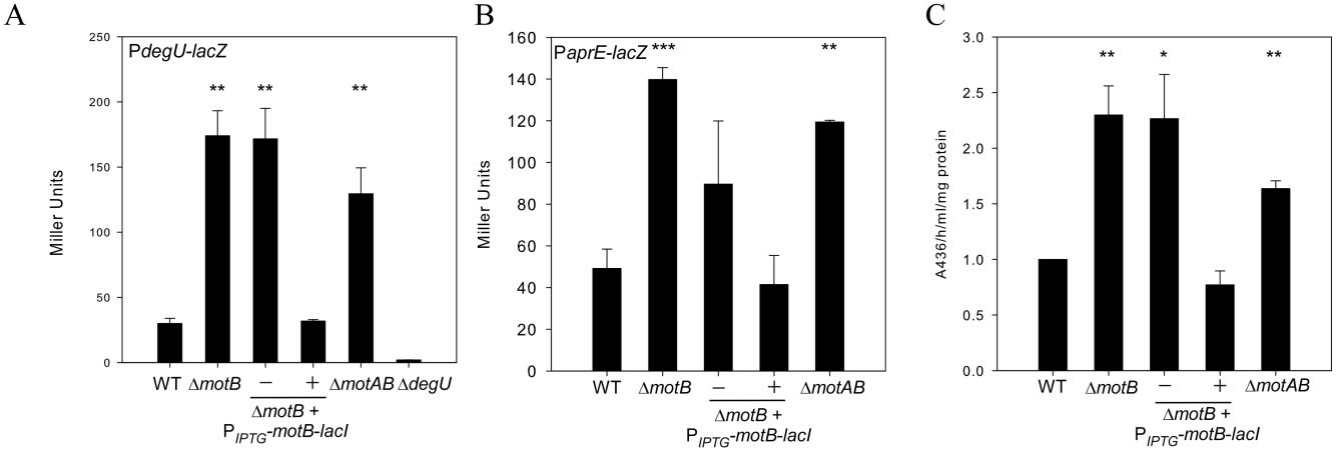
Transcription of *degU* and *aprE* is increased alongside total protease activity in Δ*motB*.A. β-Galactosidase assays of strains carrying the P*degU*–*lacZ* transcriptional reporter fusion. Strains shown are WT (wild-type NRS4351), Δ*motB* (NRS4345), *motB* + *amyE::*P_*IPTG*_-*motB*-*lacI* (NRS4396) grown in the absence or presence of 50 μM IPTG, Δ*motAB* (NRS4354) and Δ*degU* (NRS4373). All cells were collected at the onset of stationary phase.B. β-Galactosidase assays of strains carrying the P*aprE*–*lacZ* transcriptional reporter fusion. Strains shown are WT (wild-type NRS1561), Δ*motB* (NRS3440), Δ*motB* + *amyE::*P_*IPTG*_-*motB*-*lacI* (NRS3858) grown in the absence or presence of 50 μM IPTG and Δ*motAB* (NRS4093). All cells were collected at the onset of stationary phase.C. Total protease activity assays performed with the supernatant collected from cells grown in (B).Data in parts (A), (B) and (C) are plotted as the average of at least three independent replicates; in (C) data are represented as a fold change relative to the wild-type strain which was assigned value of 1. Error bars represent standard error of the mean. An asterisk denotes significance as calculated by the Student's *t*-test where * represents *P* < 0.05, ** *P* < 0.01 and *** *P* < 0.001.

To determine if DegU∼P levels were increased in the Δ*motB* strain two approaches were taken. First, transcription of *degU* was quantified upon deletion of *motB* and second, protease production was quantified as an indirect measure of DegU∼P levels. DegU∼P positively autoregulates transcription of *degU*, therefore measuring the level of activity from the *degU* promoter provides an indirect measurement of DegU∼P in the cell (Kobayashi, 2007; Veening *et al*., 2008). To this end, a P*degU–lacZ* transcriptional reporter fusion was constructed and integrated into the wild-type, Δ*motB* and Δ*motB* complemented strains. Cells were harvested from cultures grown to the onset of stationary phase, and β-galactosidase assays performed. As shown in Fig. 3A, in Δ*motB* transcription of *degU* is increased approximately fourfold when compared with the wild-type (*P* < 0.01). This effect is specific to deletion of *motB* as transcription of *degU* can be restored to wild-type levels by expression of *motB* from an IPTG-inducible promoter integrated at a non-essential locus (Fig. 3A).

To test the effect of deletion of both stator components a Δ*motAB* (NRS3744) strain was constructed. Consistent with inhibition of flagellar rotation promoting an increase in DegU∼P levels, the phenotypes reported for Δ*motB* were replicated in the double mutant strain. We first established that the non-polar *motAB* deletion strain synthesized flagella and exhibited a motility defect that could be specifically complemented by heterologous induction of *motAB* transcription using an IPTG-dependent promoter (Fig. S1A and C). Next, we examined the phenotype when grown on LB agar plates. The Δ*motAB* deletion strain exhibited a mucoid colony phenotype that could be complemented upon heterologous expression of *motAB* (Fig. S1B). Consistent with these findings, transcriptional analysis demonstrated that the *motAB* strain showed an increase in the level of *degU* expression (Fig. 3A). These data suggest that DegU∼P levels are elevated in the both Δ*motB* and Δ*motAB* strains.

High levels of DegU∼P are closely associated with exoprotease biosynthesis (Dahl *et al*., 1992; Kobayashi, 2007; Verhamme *et al*., 2007). DegU∼P positively regulates transcription of the *aprE* gene, which encodes the alkaline protease subtilisin (Mukai *et al*., 1990). We therefore hypothesized that if DegU∼P levels were high in the absence of *motB* this would correlate with an increase in *aprE* transcription and, moreover, an increase in the total protease activity in the extracellular environment. To establish if *aprE* transcription was altered in the Δ*motB* and Δ*motAB* strains, a P*aprE–lacZ* transcriptional reporter fusion was integrated at the non-essential *thrC* locus. Cells were harvested for β-galactosidase activity assays and the culture supernatant collected for measurement of extracellular protease activity (see *Experimental procedures*). Transcription of *aprE* was increased approximately threefold in the Δ*motB* (*P* < 0.001) and twofold in the Δ*motAB* (*P* < 0.01) strains when compared with the wild-type (Fig. 3B). The increase in transcription was specific as induction of *motB* transcription in the *motB* deletion strain reduced *aprE* transcription levels back to wild-type levels (Fig. 3B). In accordance with these data, total protease activity was increased by twofold upon deletion of *motB* and 1.6-fold in the Δ*motAB* strain (Fig. 3C) (*P* < 0.01).

Previous work has indicated that DegU can be phosphorylated in the absence of DegS (Kobayashi, 2007; Verhamme *et al*., 2007). To demonstrate that the upregulation in DegU∼P processes seen in the Δ*motB* strain was transmitted through DegS, the *degS* gene was disrupted in the Δ*motB* background and γ-PGA production assessed by colony morphology. As seen in Fig. 1A, the colony morphology of the Δ*motB* Δ*degS* strain reverted to that of the wild-type. Collectively these findings demonstrate that deletion of the flagellar stator gene, *motB*, causes an increase in the DegU∼P level within the population, leading to an upregulation of at least two distinct DegU∼P regulated processes. This is reliant on the presence of DegS.

### Mutation of *motB* to disturb proton flux phenocopies the Δ*motB* strain

Given the importance of MotB in the generation of torque, we hypothesized that the increase in DegU∼P regulated processes in the Δ*motB* background might be linked with the inhibition of flagellar rotation. Although the precise mechanisms by which torque is generated are not yet fully understood, it has been shown that protonation of a conserved aspartate in the MotB transmembrane domain is essential (Sharp *et al*., 1995; Zhou *et al*., 1998b). To test if disruption of proton flux through the MotAB complex, and therefore inhibition of flagellar rotation, were key to triggering an increase in DegU∼P levels, the conserved aspartate residue of *motB* (aspartate 24 in *B. subtilis*; Fig. S2A) was mutated to alanine (*motB* D^24^A) by site-directed mutagenesis. This construct was first integrated into the wild-type strain at a non-essential site on the chromosome under the control of an IPTG-inducible promoter. Upon induction of expression with 1 mM IPTG, the mutated *motB* allele conferred a dominant-negative phenotype with respect to both swarming motility (Fig. S2B) and γ-PGA production (Fig. S2D). These findings demonstrate that the MotB-D^24^A protein is synthesized and is functional as it has a dominant phenotype over the native MotB. Next, the construct was integrated into the Δ*motB* strain background. As expected, in contrast to complementation of the *motB* strain with the wild-type allele of *motB*, induction of *motB* D^24^A transcription did not restore motility to the *motB* deletion strain (Fig. S2C). Next the effect of the *motB* D^24^A mutation with regard to *degU* and *aprE* transcription, protease activity and γ-PGA biosynthesis was assessed.

**Figure 4:**
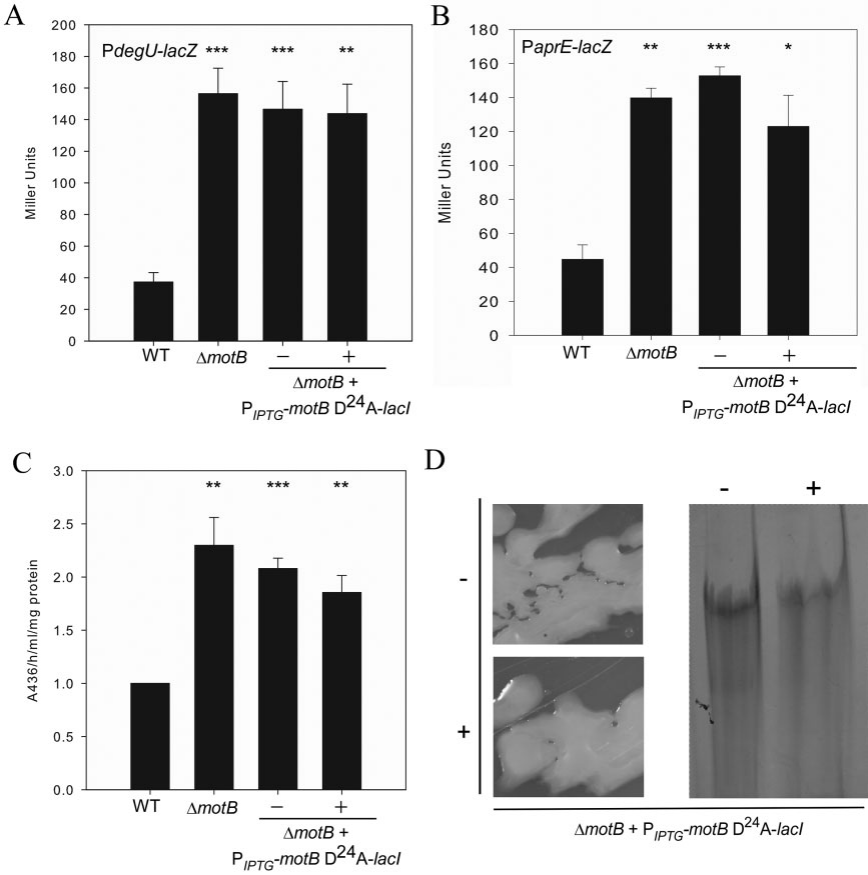
The *motB* D^24^A allele cannot complement Δ*motB*.A and B. β-Galactosidase assays of strains carrying the (A) P*degU*–*lacZ* or (B) P*aprE*–*lacZ* transcriptional reporter fusion. Strains shown are WT (wild-type NRS4351), Δ*motB* (NRS4345), *motB* + *amyE::*P_*IPTG*_-*motB*-D^24^A-*lacI* (NRS4397) grown in the absence or presence of 50 μM IPTG and Δ*degU* (NRS4373).B. β-Galactosidase assays of strains carrying the P*aprE*–*lacZ* transcriptional reporter fusion. Strains shown are WT (wild-type NRS1561), Δ*motB* (NRS3440), Δ*motB* + *amyE::*P_*IPTG*_-*motB*-D^24^A-*lacI* (NRS3870) grown in the absence or presence of 50 μM IPTG. All cells in (A) and (B) were collected at the onset of stationary phase.C. Total protease activity assays performed with supernatants collected from cells grown in (B).Data in (A), (B) and (C) are plotted as the average of at least three independent replicates. In (C) data are represented as a fold change relative to the wild-type strain which was assigned value of 1. Error bars represent standard error of the mean. An asterisk denotes significance as calculated by the Student's *t*-test where * represents *P* < 0.05; **P < 0.01; and *** *P* < 0.001.D. Colony morphology of Δ*motB* + *amyE::*P_*IPTG*_-*motB*-D^24^A-*lacI* (NRS3870) grown on LB agar in the absence and presence of 50 μM IPTG. SDS-PAGE analysis of γ-PGA collected from cultures of Δ*motB* + *amyE::*P_*IPTG*_-*motB*-D^24^A-*lacI* (NRS3870) grown in the absence or presence of 50 μM IPTG.

It was determined that induction of *motB* D^24^A expression was unable to complement Δ*motB* with respect to both *degU* and *aprE* transcription, which remained fivefold and threefold higher than in the wild-type respectively (Fig. 4A and B). Similarly protease activity was maintained at a higher level in the presence of *motB* D^24^A (Fig. 4C). γ-PGA production was confirmed based on the mucoid colony morphology and analysis of exopolymers extracted from culture supernatant (Fig. 4D). These data support the hypothesis that perturbation of proton flux, and therefore flagellar rotation, is necessary to trigger an increase in DegU∼P activity.

### Engaging the flagellar clutch causes an increase in *degU* transcription

To assess if the increase in DegU∼P regulated processes was specific to deletion or mutation of the flagellar stator genes the effect of perturbing flagellar rotation by an alternative genetic means was tested. The *epsE* gene encodes a bi-functional protein, EpsE, that can act as (i) a flagellar clutch to disable flagellar rotation and (ii) a glycosyltransferase enzyme required for the formation of robust biofilms (Blair *et al*., 2008; Guttenplan *et al*., 2010). Overexpression of *epsE* under the control of an IPTG-inducible promoter inhibits motility by interaction with FliG, thereby disengaging the rotor from the stator (Blair *et al*., 2008; Guttenplan *et al*., 2010). To test if inhibition of flagellar rotation by overexpression of *epsE* would also trigger an increase in DegU∼P levels, the coding region of *epsE* was integrated at a non-essential locus on the chromosome under the control of an IPTG-inducible promoter. To ensure that any effect on DegU∼P was specific to the clutch activity of *epsE* and not due to the glycosyltransferase function, site-directed mutagenesis was used to mutate aspartate 94 to alanine (*epsE* D^94^A) to yield a protein variant that retained clutch activity but lost glycosyltransferase functionality. Simultaneously, lysine 106 was mutated to glutamic acid (*epsE* K^106^E) to yield a protein variant that lost clutch activity but possessed glycosyltransferase activity. Both single amino acid mutations have been previously characterized by Guttenplan *et al*. (2010).

**Figure 5:**
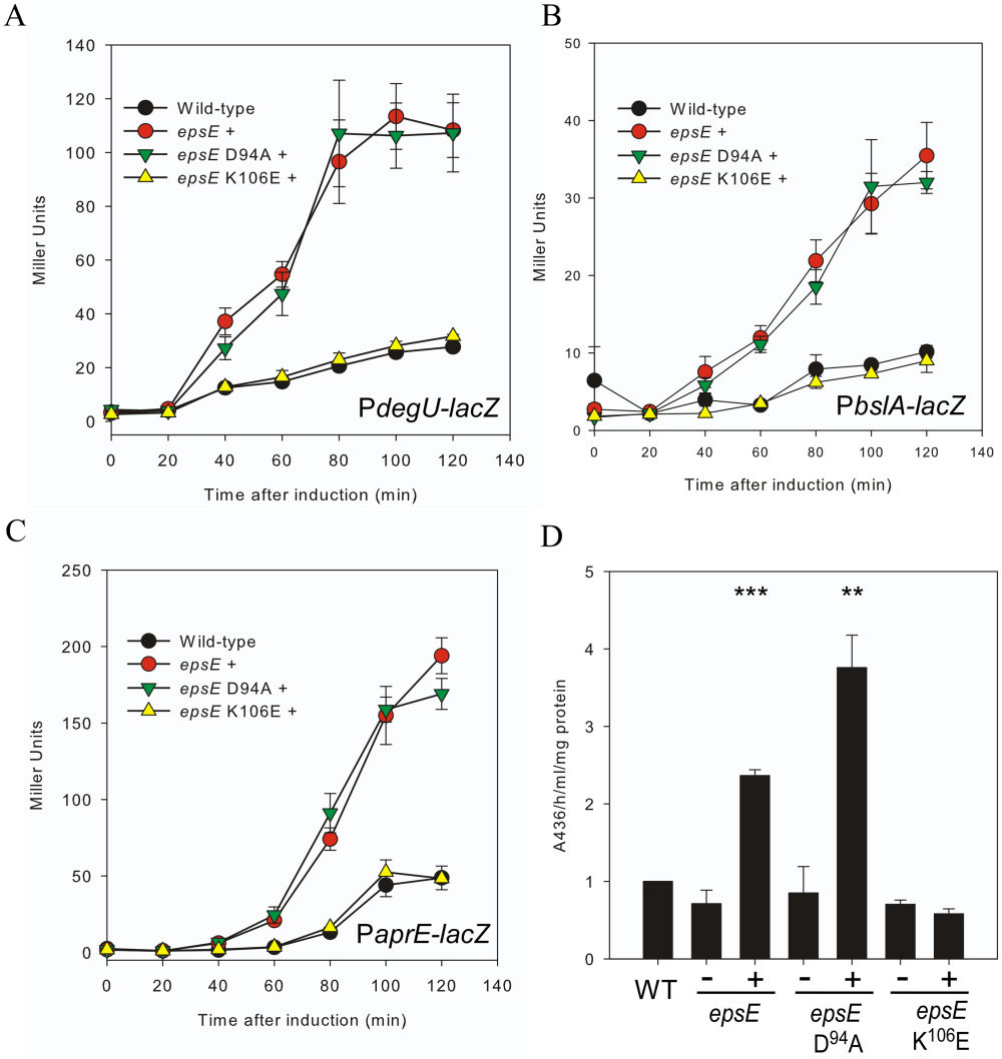
Engaging the flagellar clutch causes an increase in DegU∼P levels.A–C. β-Galactosidase assays of strains carrying the (A) P*degU*–*lacZ*, (B) P*bslA*–*lacZ* or (C) P*aprE*–*lacZ* transcriptional reporter fusion. Cells were grown to 0.5 OD_600_ and induced with 1 mM IPTG (final concentration). Strains shown are: (A) wild-type (NRS4351), *epsE* + (P_*IPTG*_-*epsE*-*lacI* (NRS4374)), *epsE* D^94^A + (P_*IPTG*_-*epsE*-D^94^A-*lacI* (NRS4392)) and *epsE* K^106^E (P_*IPTG*_-*epsE*-K^106^E-*lacI* (NRS4394)); (B) Wild-type (NRS2052), P_*IPTG*_-*epsE*-*lacI* (NRS4405), P_*IPTG*_-*epsE*-D^94^A-*lacI* (NRS4406) and P_*IPTG*_-*epsE*-K^106^E-*lacI* (NRS4407); (C) Wild-type (NRS1561), P_*IPTG*_-*epsE*-*lacI* (NRS4345), P_*IPTG*_-*epsE*-D^94^A-*lacI* (NRS4393) and P_*IPTG*_-*epsE*-K^106^E-*lacI* (NRS4395). Data shown in (A), (B) and (C) are plotted as the average of at least three independent replicates. Error bars represent standard error of the mean.D. Total protease activity assays performed with supernatants collected from cells grown in (C) after 120 min of induction in the absence or presence of 1 mM IPTG. Data are plotted as the average of at least three independent replicates and are represented as a fold change relative to the wild-type strain (WT) which was assigned value of 1. Error bars represent standard error of the mean. An asterisk denotes significance as calculated by the Student's *t*-test, where ** represents *P* < 0.01; and *** *P* < 0.001.

First, the motility phenotypes of these strains were assessed to ensure that the point mutations introduced functioned as expected. As shown in Fig. S3A, induction of either the *epsE* WT or *epsE* D^94^A coding regions inhibited swarming motility, while induction of the *epsE* K^106^E coding region in the otherwise wild-type strain had no impact on swarming motility as compared with NCIB3610. As these phenotypes are entirely in line with the previous report (Guttenplan *et al*., 2010), we proceeded to assess the effect of these mutations on *degU* transcription. The strains were grown to mid-exponential phase, at which point expression of *epsE* (or the mutant alleles) was induced by the addition of IPTG. Samples were collected over time to assess β-galactosidase activity. Induction of *epsE* WT or *epsE* D^94^A expression resulted in a fourfold increase in *degU* transcription by comparison with the wild-type, while induction of *epsE* K^106^E phenocopied the wild-type (Fig. 5A and Fig. S3B).

### Engaging the flagellar clutch causes an increase in DegU∼P regulated processes

To test if the upregulation of *degU* transcription observed upon induction of *epsE* translated to an increase in DegU∼P-regulated processes, transcription of the *bslA* gene was measured. The *bslA* promoter is the main target of DegU∼P during biofilm formation by *B. subtilis* (Kobayashi, 2007; Verhamme *et al*., 2009; Ostrowski *et al*., 2011). Therefore, it was hypothesized that an increase in DegU∼P levels by inhibition of flagellar rotation would result in increased *bslA* transcription. A P*bslA*–*lacZ* transcriptional reporter fusion was integrated into the *epsE* WT, D^94^A and K^106^E strains and activity measured over time by β-galactosidase assays. A similar trend to that reported for *degU* transcription was observed. Induction of expression of *epsE* WT or *epsE* D^94^A resulted in a threefold increase in transcription by comparison with the wild-type (Fig. 5B and Fig. S3C). As predicted from the *degU* transcription analysis results, induction of *epsE* K^106^E phenocopied the wild-type strain.

The effect of overexpression of *epsE* on *aprE* transcription and total protease activity was then tested. As seen for *degU* and *bslA* transcription, *aprE* transcription was increased threefold upon inhibition of flagellar rotation (Fig. 5C and Fig. S3D). In accordance with the *aprE* transcription analysis data, 120 min after induction of *epsE* or *epsE* D^94^A, total protease activity was twofold higher than the wild-type levels, while the protease activity of the *epsE* K^106^E strain was not significantly different from the wild-type (Fig. 5C). Consistent with DegU∼P directing both *bslA* transcription (Kobayashi, 2007; Verhamme *et al*., 2009) and exoprotease production (Mukai *et al*., 1990), we noted that the level of *degU* transcription started to increase 20 min after IPTG induction of *epsE* transcription, whereas both *bslA* and *aprE* transcription began to rise 40 min post induction (compare Fig. 5A with Fig. 5B and C). These data demonstrate that *bslA* and *aprE* are only transcribed after the level of DegU∼P increases. It is important to note that at this point the transcriptional repressor AbrB will have been removed from the promoter elements (Olmos *et al*., 1996; Verhamme *et al*., 2009).

**Figure 6:**
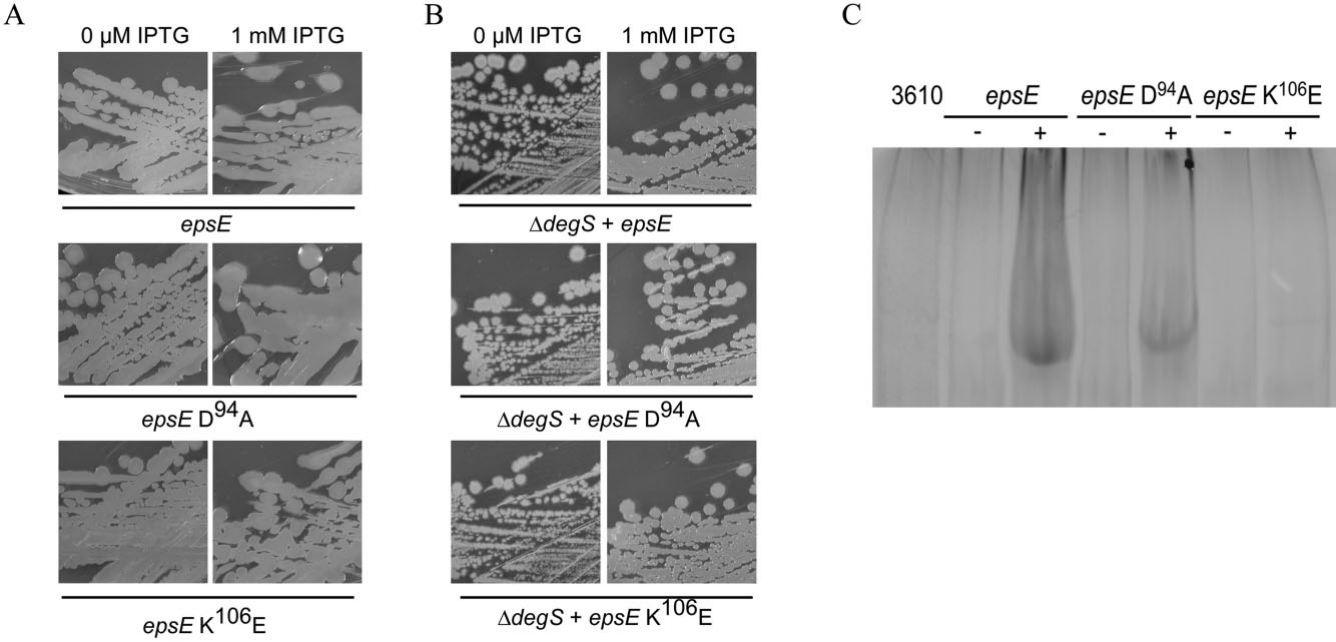
Engaging the flagellar clutch triggers an increase in γ-PGA production that requires DegS.A. Colony morphology of P_*IPTG*_-*epsE*-*lacI* (NRS4085), P_*IPTG*_-*epsE*-D^94^A-*lacI* (NRS4388) and P_*IPTG*_-*epsE*-K^106^E-*lacI* (NRS4389) in the absence and presence of 1 mM IPTG after growth overnight at 37°C.B. Colony morphology of P_*IPTG*_-*epsE*-*lacI* + *degS::cml* (NRS4399), P_*IPTG*_-*epsE*-D^94^A-*lacI* + *degS::cml* (NRS4400) and P_*IPTG*_-*epsE*-K^106^E-*lacI* + *degS::cml* (NRS4401) in the absence and presence of 1 mM IPTG after growth overnight at 37°C.C. SDS-PAGE of γ-PGA collected from cultures of 3610 (wild-type NRS1561), P_*IPTG*_-*epsE*-*lacI* (NRS4345) and P_*IPTG*_-*epsE*-D^94^A-*lacI* (NRS4393) and P_*IPTG*_-*epsE*-K^106^E-*lacI* (NRS4395), grown in the absence or presence of 1 mM IPTG, at the onset of stationary phase.

γ-PGA production was then assessed. Induction of either *epsE* WT or *epsE* D^94^A expression overnight on LB-agar plates containing 1 mM IPTG yielded a mucoid colony phenotype (Fig. 6A). The mucoid phenotype was not seen when IPTG was lacking or when either the wild-type or *epsE* K^106^E expression strain was examined (Fig. 6A). Consistent with the colony phenotypes, γ-PGA was extracted from the culture supernatant and analysed by SDS-PAGE for both the *epsE* WT and *epsE* D^94^A expression strains (Fig. 6C). To determine if the γ-PGA production, and hence DegU∼P levels, was reliant on input from DegS, the *degS* gene was disrupted in the *epsE* WT, *epsE* D^94^A and *epsE* K^106^E expression strains and γ-PGA production monitored using colony phenotype in the absence and presence of IPTG. A dry, flat colony morphology was observed for all strains carrying the *degS* disruption upon heterologous expression of *epsE* and the mutant *epsE* alleles with IPTG (Fig. 6B). Taken together, these data indicate that inhibition of flagellar rotation by EpsE results in an increase in DegU∼P levels, which is reflected by an upregulation of *bslA* transcription, exoprotease activity and γ-PGA production. As proven for the *motB* strain (Fig. 1), DegS is responsible for increasing the levels of DegU∼P upon induction of EpsE. Thus the signal generated by inhibition of flagellar rotation when EpsE is present is likely to be the same as when flagellar rotation is inhibited due to mutation or disruption of MotB.

### Tangling of flagella increases DegU∼P activity

**Figure 7:**
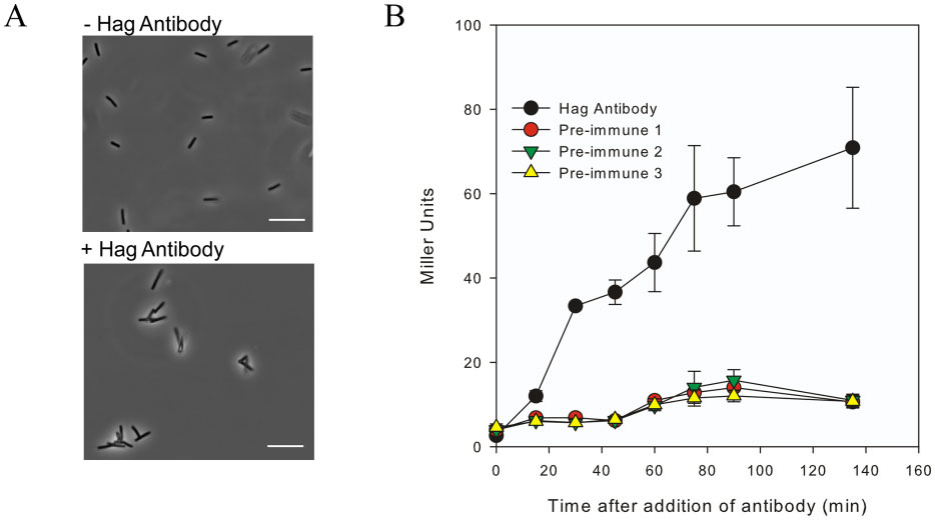
Tethering the flagella triggers an increase in *degU* transcription.A. Micrographs of cells containing the P*degU*–*lacZ* transcriptional reporter fusion (NRS4351) grown presence or absence of Hag antibody in sera. Micrographs are static images taken from movies filmed 5 min after the addition of antibody to the culture (see Movies S1 and S2). Scale bars represent 100 pixels.B. β-Galactosidase assays of the wild-type strain carrying the P*degU*–*lacZ* transcriptional reporter fusion (NRS4351) grown in the presence of a 1:20 dilution of either pre-immune sera or the Hag-specific antibody. Three independent pre-immune sera were used as controls. Data are plotted as the average of at least three independent replicates. Error bars represent standard error of the mean.

Data presented thus far indicate that inhibition of flagellar rotation by genetic manipulation activates the DegS–DegU two-component signal transduction system. We next used a non-genetic method to block flagellar rotation (Meister *et al*., 1987). An antibody raised against the flagellar filament protein, Hag, was used to tangle flagella (see Fig. 7A and Movies S1–S4) and the impact on *degU* transcription measured. Prior to this we used Western blot analysis to check the specificity of the Hag antibody (Fig. S4). To ensure that any effects seen were specific to the Hag antibody, and not due to off-target effects of the serum, three independent pre-immune sera were used as controls. The wild-type strain carrying the P*degU–lacZ* transcriptional reporter fusion was grown in liquid culture to mid-exponential phase at which point either pre-immune sera or the Hag antibody in sera was added (1:20 dilution). Swimming motility was immediately checked by real-time live single cell microscopy (see Fig. 7A and Movies S1–S4) and samples were collected over time for β-galactosidase assays. Analysis demonstrated that transcription from the *degU* promoter was upregulated only 15 min after addition of the antibody, and increased fourfold by comparison with the pre-immune sera controls after 30 min (Fig. 7B) (*P* < 0.001). This trend of increased transcription was maintained over the course of the experiment (Fig. 7B). To further validate these data, exoprotease activity assays were undertaken with samples collected at the end of each time-course. The mean level of exoprotease activity for the three pre-immune sera controls was calculated and compared with that of the experimental sample incubated with Hag anti-sera. Analysis demonstrated a statistically significant (*P* = 0.01) 13-fold increase in exoprotease activity upon inhibition of flagellar rotation by tangling of the flagella. The mean level of protease activity in the presence of sera alone was 0.004 ± 0.001 Δ*A*_436_ h^−1^ ml^−1^ per mg of total protein compared with 0.05 ± 0.001 Δ*A*_436_ h^−1^ ml^−1^ per mg of total protein in the presence of the sera containing the anti-hag antibody. Collectively, these data unequivocally demonstrate that inhibition of rotation of the *B. subtilis* flagellum either genetically or mechanically results in an increase in *degU* transcription and DegU∼P regulated processes, findings that are consistent with the activation of the sensor kinase, DegS.

## Discussion

In the natural environment bacteria predominantly live adhered to a surface as part of a biofilm (Costerton *et al*., 1995; Vlamakis *et al*., 2013). However, how a motile cell is able to sense and respond to the presence of a surface is poorly understood. Here, we show that inhibition of flagellar rotation acts as a signal to trigger an increase in the level of DegU∼P via the sensor kinase DegS. This is a regulatory pathway that is needed for biofilm formation by the Gram-positive bacterium *B. subtilis* (Stanley and Lazazzera, 2005; Kobayashi, 2007; Verhamme *et al*., 2007). These findings clearly demonstrate that *B. subtilis* can effectively use the flagellum for both signal transduction purposes as well as a mechanical device for propulsion. These findings add to the small but growing body of evidence indicating that the flagellum is utilized by the cell to explore and respond to external stimuli in diverse bacterial species (Belas and Suvanasuthi, 2005; Wang *et al*., 2005; Gode-Potratz *et al*., 2011; Friedlander *et al*., 2013). We propose that inhibition of flagellar rotation would occur due to physical contacts with a surface, and that the consequential activation of a signal transduction pathway may present a mechanism by which flagellated organisms detect and respond to a surface.

### Activation of the DegS–DegU two-component regulatory system

Previous work has highlighted a role of DegU∼P in controlling flagellar assembly (Amati *et al*., 2004; Hsueh *et al*., 2011). Indeed it has been shown that DegU is preferentially activated in genetic backgrounds that promote cell chaining, with further experiments tentatively suggesting that DegU might play a role in sensing the status of flagellar assembly (Hsueh *et al*., 2011). The data presented herein demonstrate that the DegS–DegU two-component regulatory system is activated when the flagellum stops rotating. Therefore DegU∼P has a role both in flagellar biosynthesis and in responding to signals inputted by the flagellum. Data demonstrating that inhibition of flagellar rotation acts as a trigger to activate the DegS–DegU two-component system allows the *B. subtilis* flagellum to be classified as a mechanosensor that controls bacterial cell behaviour. To the best of our knowledge this is the first example of a classical two-component system being activated in response to a mechanical signal: namely inhibition of flagellar rotation. We suggest that repression of flagellar rotation occurs when the flagellum encounters a surface; a process that is mimicked in our study by flagella tangling experiments (Fig. 7).

### Surface sensing by the flagellum

Our findings add *B. subtilis* to the small list of microorganisms for which surface sensing has been linked with downstream alterations in gene transcription and protein synthesis (McCarter *et al*., 1988; Kawagishi *et al*., 1996). For example, transcriptomic and proteomic screens have shown that genes and proteins are differentially regulated between liquid and surface grown bacteria (Kim and Surette, 2004; Wang *et al*., 2004). Moreover, in *Vibrio parahaemolyticus*, which possesses a dual flagellar system, it has been shown that slowing the rotational speed of the polar flagellum triggers gene expression changes that result in the transcription of genes essential for the synthesis of lateral flagella (McCarter *et al*., 1988; Kawagishi *et al*., 1996). Furthermore, inhibition of rotation of the polar flagellum also impacts genes associated with cyclic-di-GMP signalling and virulence, thereby suggestive of a global role for flagellar mediated surface sensing in controlling bacterial cell physiology (Gode-Potratz *et al*., 2011).

### The transition from motility to attachment

The attachment of bacteria to a surface is the first step in the formation of a biofilm, where the initial stage of adhesion is often mediated by flagella-or pili-based motility (O'Toole *et al*., 2000). Indeed, several studies have identified flagella, or flagellar motility, as a key aspect of biofilm development and biofilm ‘microanatomy’ (O'Toole and Kolter, 1998; Pratt and Kolter, 1998; Watnick and Kolter, 1999; Lemon *et al*., 2007; Friedlander *et al*., 2013; Serra *et al*., 2013). The subsequent transition to persistent adhesion is often mediated by polysaccharides (Watnick and Kolter, 2000). In *Caulobacter crescentus* a direct link between surface-sensing by the flagellum and irreversible adhesion has been shown, where upon reaching the surface, pili-dependent inhibition of flagellum rotation stimulates concomitant synthesis of the holdfast, promoting permanent attachment (Li *et al*., 2012). Moreover, exopolysaccharides have been shown to ‘wheel-lock’ flagellar rotation resulting in co-ordination of motility inhibition and stimulation of biofilm formation (Zorraquino *et al*., 2013). Alternatively, or indeed additionally, flagellar brake or clutch activity can play a role in the transition between motility and biofilm formation to ensure separation of these mutually exclusive cell behaviours (Blair *et al*., 2008; Pilizota *et al*., 2009; Boehm *et al*., 2010; Paul *et al*., 2010). This can be exemplified by the *B. subtilis* flagellar clutch, EpsE that was utilized in this study. It is of interest to note that EpsE is encoded within the *epsA-O* operon that is required for the synthesis of one of the major structural components of the biofilm, the exopolysaccharide. EpsE therefore links inhibition of motility to exopolysaccharide production and biofilm formation (Blair *et al*., 2008; Guttenplan *et al*., 2010; Guttenplan and Kearns, 2013). Here we provide evidence that induction of EpsE will reinforce biofilm formation through increased biosynthesis of BslA (Fig. 5B), a bacterial hydrophobin that coats the *B. subtilis* biofilm (Kobayashi and Iwano, 2012; Hobley *et al*., 2013) and functions synergistically with the exopolysaccharide and TasA amyloid fibres to facilitate assembly of the biofilm matrix (Ostrowski *et al*., 2011). Proteins functionally similar to EpsE have been classed as flagellar ‘brakes’ in *E. coli* (Boehm *et al*., 2010; Paul *et al*., 2010) and *Rhodobacter sphaeroides* (Pilizota *et al*., 2009). Therefore, the ability of a single protein to inhibit flagellar rotation appears to be a general mechanism used by bacteria to facilitate the transition to a sessile state.

### Concluding remarks

There are, of course, outstanding questions that follow our discovery; the first being how a slowing or lack of flagellar rotation is sensed. There are several mechanisms by which this might occur. One possibility is that DegS, as a cytoplasmic sensor kinase, interacts with components of the flagellar motor when rotation is stopped. This postulated mechanism is similar to that proposed for the cyclic-di-GMP (c-di-GMP)-binding protein, YcgR, which interacts with the flagellar motor proteins FliG, FliM (Paul *et al*., 2010) and MotA (Boehm *et al*., 2010), when c-di-GMP levels are high resulting in the inhibition of motility. Consistent with this, in *Pseudomonas aeruginosa* the chemotaxis-like Wsp signal transduction system, which is essential for biofilm formation, has been shown to produce c-di-GMP in response to surface growth (Guvener and Harwood, 2007; O'Connor *et al*., 2012). A second possible mechanism may involve the stator-associated transmembrane protein, FliL. Previous studies have identified FliL as a possible intermediary between the inhibition of rotation and signal transduction (Belas and Suvanasuthi, 2005; Cusick *et al*., 2012). Moreover, recent studies in different bacterial species have identified several roles for FliL in bacterial motility and surface-sensing (Lee *et al*., 2013). For example, FliL has been shown to be associated with (Motaleb *et al*., 2011) and enhance the function of the flagellar stator (Suaste-Olmos *et al*., 2010), with evidence now suggesting that overexpression of FliL alongside MotAB is sufficient to overcome surface friction associated with swarming on hard agar (Partridge and Harshey, 2013). Therefore, given that the function of FliL does not appear to be strictly conserved between bacterial species it will be of interest to determine if FliL is also a key player in surface-sensing and flagellar rotation in *B. subtilis*. Alternatively, we cannot exclude the possibility that DegS might directly sense changes in the intracellular environment that are triggered by a lack of flagellar rotation, such as ion flux (Kawagishi *et al*., 1996) or potentially energy status (Watson and Fedor, 2012). Intriguingly, recent work in *E. coli* has identified the flagellar stator, not the flagellar filament, as a mechanosensor that is able to remodel itself in a load-dependent manner (Lele *et al*., 2013). It is therefore possible that the increase in the number of stators required to drive flagellar torque under high loads might itself act as a signal to impact downstream signalling pathways. It will be of interest in the future to determine the underlying molecular detail of DegS activation, and moreover to clarify if our hypothesis that the flagellum acts as a mechanosensor to allow a sessile lifestyle to be adopted by other flagellated bacterial species holds true.

## Experimental procedures

### Growth conditions and strain construction

*Escherichia coli* and *B. subtilis* strains were routinely grown in Luria–Bertani (LB) broth (10 g NaCl, 5 g yeast extract, 10 g tryptone per litre) or on LB plates supplemented with 1.5% select agar (Invitrogen) at 37°C unless otherwise stated. When appropriate, isopropyl β-d-1-thiogalactopyranoside (IPTG) was added at the indicated concentrations. *E. coli* strain MC1061 [*F*'*lacIQ lacZM15 Tn10* (*tet*)] was used for the routine construction and maintenance of plasmids. When required, antibiotics were used at the following concentrations: 100 μg ml^−1^ ampicillin, 100 μg ml^−1^ spectinomycin, 1 μg ml^−1^ erythromycin and 25 μg ml^−1^ lincomycin. Strains were constructed using standard protocols. Phage transductions were carried out as previously described (Verhamme *et al*., 2007). A full list of strains used in this study is provided in Table S1.

### Construction of deletion strains

To construct the in-frame deletion of *motB* an approach similar to that previously described was used (Kiley and Stanley-Wall, 2010). The upstream region of *motB* was amplified from genomic DNA using primers NSW874 and NSW875, purified and digested with BamHI and XbaI, using the restriction sites engineered into the primers and ligated into pUC19 (Vieira and Messing, 1982) cut the same to yield pNW651. The downstream region of *motB* was amplified using primers NSW876 and NSW877, purified, and digested with XbaI and BamHI, using the restriction sites engineered into the primers and ligated into pUC19 cut the same to yield pNW652. The upstream and downstream regions of *motB* were released from pNW651 and pNW652 with BamHI and XbaI and XbaI and EcoRI, respectively, and ligated into pUC19 cut with BamHI and EcoRI to produce plasmid pNW653. The Δ*motB* region was then cut from pNW653 with BamHI and EcoRI and ligated into pMAD cut the same (Arnaud *et al*., 2004). Strain NRS3494 (NCIB3610 Δ*motB*) was generated by integration and curing of the region contained in pNW654 in strain NCIB3610. An in-frame deletion of *motAB* was constructed in a similar manner. The upstream region of *motA* was amplified from genomic DNA using primers NSW965 and NSW966, purified and digested with BamHI and XbaI, using restriction sites engineered into the primers. The downstream region of *motB* was excised from pNW652 with XbaI and EcoRI. The upstream region of *motA* and downstream region of *motB* were ligated into pUC19 cut with BamHI and EcoRI to yield pNW1019. The Δ*motAB* region was then cloned into pMAD cut with BamHI and EcoRI to yield pNW1021. Strain NRS3744 (NCIB3610 Δ*motAB*) was generated by integration and curing of the region contained in pNW1021 in strain NCIB3610. All primers and plasmids used in this study are listed in Tables S2 and S3.

### Reverse transcription (RT)-PCR

RNA was isolated from the following strains grown to mid-exponential phase: NCIB3610 (wild-type), NRS3494 (Δ*motB*) and NRS3775 (Δ*motB* + P*_spankhy_-motB-lacI*) with or without 50 μM IPTG. RNA isolation was carried out as described previously (Kiley and Stanley-Wall, 2010) using the RiboPure Bacteria RNA Isolation Kit (Ambion), according to manufacturer's instructions. cDNA was synthesized using random hexamers and subsequently treated with Rnase H for 20 min at 37°C. To amplify internal gene products the following primer pairs were used: NSW1474 and NSW1475 (*pgsB*), NSW1604 and NSW1605 (*pgdS*) and DEN5 and DEN7 (16S rRNA).

### Motility assays

Swimming and swarming analyses were performed as described before (Verhamme *et al*., 2007) using low-salt LB (5 g NaCl, 5 g yeast extract, 10 g tryptone per litre) supplemented with 0.4% or 0.7% Bacto agar (Invitrogen) respectively. Plates were incubated at 37°C and the extent of swimming or swarming noted at defined intervals.

### Whole-cell analysis of Hag

Proteins were collected from planktonic cultures grown to mid-exponential phase. Briefly, cells were harvested by centrifugation at 4700 *g.* Cells were suspended in 1× Bugbuster Master Mix (Novagen) and lysed according to manufacturer's instructions. Seven micrograms of proteins were resolved by SDS-PAGE and stained with Coomassie Brilliant Blue. Hag protein was identified by 1D SDS-PAGE analysis of the total cellular proteins by comparison with the Δ*hag* strain (DS1677). The protein identity was confirmed by mass spectrometry (FingerPrints Proteomics and Mass Spectrometry Facility, University of Dundee) (Diethmaier *et al*., 2011).

### Inhibition of flagellar rotation with an anti-Hag antibody

A wild-type strain carrying the P*degU–lacZ* transcriptional reporter fusion (NRS4351) was grown to OD_600_ of 0.4 in LB prior to addition of a 1 in 20 dilution of Hag antibody (Prof. Kursad Turgay) or pre-immune sera. To check the motility of the cells, a small sample of each culture was imaged by microscopy at each time point. A thin channel was generated between a glass slide and the coverslip using double sided sticky tape. Cells were injected into the viewing chamber and visualized using a Zeiss Axio10 Imager.M10 under a Zeiss 40× EC Plan-NEO FLUAR objective and recorded using the high speed digital recorder function in the Zen lite software (Zeiss). Samples (0.5 ml) were collected by centrifugation over the course of the experiment, frozen at −20°C and later analysed by β-galactosidase assay.

### β-Galactosidase assays

The β-galactosidase activity of strains harbouring *lacZ* promoter reporter fusions was measured as previously described (Verhamme *et al*., 2007; 2009). The values presented are the average β-galactosidase activities in Miller Units (Miller, 1972) determined from at least three independent samples. Error bars represent the standard error of the mean.

### Protease plate assays

Analysis of protease production was carried out as previously described (Verhamme *et al*., 2007). Briefly, secreted protease production was analysed using LB agar plates supplemented with 1.5% (w/v) milk. *B. subtilis* cultures were grown to mid-late exponential phase in LB and 10 μl of culture spotted on to each plate (containing IPTG as required) and incubated at 37°C for 18 h prior to being photographed.

### Secreted protease activity assay

Culture samples were collected by centrifugation (17 000 *g* for 5 min) after which the supernatant was removed and stored at −20°C until use. To determine extracellular protease activity the azocasein assay (Braun and Schmitz, 1980) was performed. A 150 μl aliquot of thawed supernatant was mixed with 500 μl of 2% w/v azocasein (Sigma), along with 100 μl of Tris-HCl (pH 8.0) and 650 μl of ddH_2_O. A blank sample was prepared containing ddH_2_O in the place of the supernatant and a media only control sample containing LB in the place of the supernatant was also prepared. The samples were incubated for 1 h at 30°C, after which 375 μl of 14% v/v perchloric acid was added to stop each reaction. The samples were centrifuged (17 000 *g* for 5 min) and 750 μl of the supernatant was mixed directly in a cuvette with 75 μl of 10 M NaOH and the absorbance at 436 nm was measured using a spectrophotometer. The background activity of the medium-only control was subtracted and activity was calculated as Δ*A*_436_ h^−1 ^ml^−1^ per mg of total protein.

### γ-PGA isolation

The method used for γ-PGA isolation was adapted from Stanley and Lazazzera, (2005). Briefly, cells were grown overnight on LB lawn plates, collected in 5 ml LB and diluted to an OD_600_ of 0.01. Cells were grown to stationary phase in 25 ml LB and harvested by centrifugation. A total of 10 ml of the culture supernatant was retained and the cell pellet suspended in 2.5 ml of 0.14 mM NaCl. The cells were again harvested by centrifugation and the supernatant from the wash added to the previously collected supernatant. The combined supernatants were brought to pH 2.0 with concentrated sulphuric acid and incubated at 4°C overnight. To precipitate γ-PGA, 40 ml 100% ethanol was added to the supernatant and the sample incubated at −20°C for a minimum of 10 min. The γ-PGA was harvested by centrifugation and the resulting pellet suspended in 1 ml 10 mM Tris-HCl pH 8.0 and concentrated in a vacuum concentrator. The resulting γ-PGA pellet was suspended in 200 μl 10 mM Tris-HCl pH 8.0 and analysed by SDS-PAGE. Gels were stained with 0.5% methylene blue in 3% acetic acid for 30 min and de-stained in H_2_O.
