# Supplementary material for: A mechanical signal transmitted by the flagellum controls signalling in Bacillus subtilis
Source: Mol Microbiol. 2013 Aug 14;90(1):6–21. doi: 10.1111/mmi.12342 (PMC3963450; doi:10.1111/mmi.12342)
Supplement: Supplementary file 5 [file mmi0090-0006-sd5.pdf]

Supplemental Material

A mechanical signal transmitted by the flagellum controls signalling in *Bacillus subtilis*

Lynne S. Cairns [1], Victoria L. Marlow [1], Emma Bissett [1], Adam Ostrowski [1], and Nicola R. Stanley-Wall [1]

[1] Division of Molecular Microbiology, College of Life Sciences, University of Dundee, Dundee, UK DD1 5EH

*Running Title: Flagella mediated signal transduction

To whom correspondence should be addressed:

Nicola R. Stanley-Wall

Division of Molecular Microbiology, College of Life Sciences, University of Dundee, Dundee DD1 5EH.

Email: n.r.stanleywall@dundee.ac.uk Tel: +44(0)1382 385136; Fax: +44(0)1382 388216;

Keywords: *Bacillus subtilis*, motility, rotation, flagellum, signal transduction, response regulator

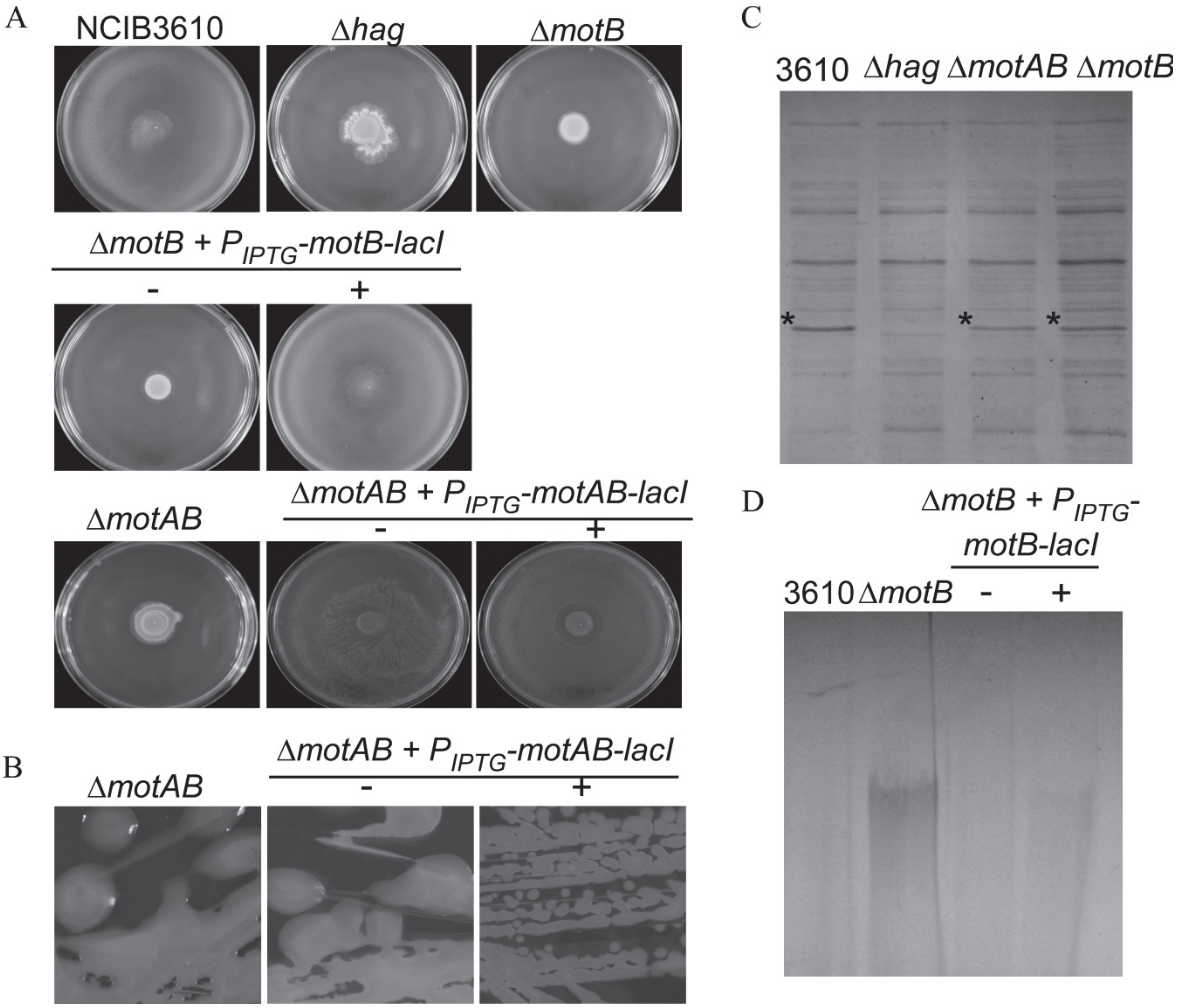

Figure S1 The Δ*motB* strain can be complemented by *motB,* but not by *motB* D^{24}A. (A) Photographs of swarm expansion assay plates taken after 6 hours incubation at 37 °C. Strains shown are NCIB3610 (wild-type), Δ*hag* (DS1677), Δ*motB* (NRS3494), Δ*motB +* P_{IPTG}-*motB-lacI* (NRS3775) grown in the absence and presence of 50 µM IPTG, Δ*motAB* (NRS3744) and Δ*motAB +* P_{IPTG}-*motAB-lacI* (NRS4387) grown in the absence and presence of 50 µM IPTG **(B)** Colony morphology of Δ*motAB* (NRS3744) and Δ*motAB +* P_{IPTG}-*motAB-lacI* (NRS 4387) grown overnight at 37°C on LB agar in the absence and presence of 1 mM IPTG. **(C)** Coomassie stained SDS-PAGE analysis examining Hag polymerisation for strains, NCIB3610 (3610), Δ*hag* (NRS2253), Δ*motAB* (NRS3744) and Δ*motB* (NRS3494). The Hag protein (as identified by mass spectrometry analysis) is indicated by the presence of an asterix. **(D)** SDS-PAGE of γ-PGA collected from cultures of NCIB3610, Δ*motB* (NRS3494) and Δ*motB +* P_{IPTG}-*motB-lacI* (NRS3775) grown to the onset of stationary phase.

A

```
E.      MKNQAHPIIVVKRRKAKSHGAAHGSWKIAYADFMTAMMAFFLVMWLISISSPKELIQIAE
B.      --------MARKKKKKHEDEHVDESWLVPYADILTLLLALFIVLYASSSIDAAKFQMLSK
                :. *::* :..  .. ** : ***::* ::*:*:*::  *  .  ::  :::

E.      YFRTPLATAVTGGDRISNSESPIPGGGD--DYTQSQG-EVNKQPNIEELKKRMEQSRLRK
B.      SF----NEVFTGGTGVLDYSSVTPPENESDGIDEVKKEKEEKEKNKKEKEKAADQEELEN
         *      ..***  : : .*  *   :     : :  : :*: * :* :*  :*..*.:

E.      LRGDLDQLIESDPKLRALRPHLKIDLVQEGLRIQIIDSQNRPMFRTGSADVEPYMRDILR
B.      VKSQVEKFIKD----KKLEHQLETKMTSEGLLITIKDS---IFFDSGKATIRKEDVPLAK
        ::.:::::*:.    : *. :*: .:..*** * * **    :* :*.* :.     : :

E.      AIAP-VLNGIPNRISLSGHTDDFPYASGEKGYSNWELSADRANASRRELMVGGLDSGKVL
B.      EISNLLVINPPRNIIISGHTDNMPIKNSE-FQSNWHLSVMRAVNFMGLLIENPKLDAKVF
         *:  ::   *..* :*****::*  ..*   ***.**. **      *:     ..**:

E.      RVVGMAATMRL--SDRGPDDAVNRRISLLVLNKQAEQAILHENAESQNEPVSALEKPEVA
B.      SAKGYGEYKPVASNKTAEGRSKNRRVEVLILPRGAAET---------NEK----------
         . * .    :  .. .   : ***:.:*:* : * ::         **

E.      PQVSVPTMPSAEPR
B.      --------------
```

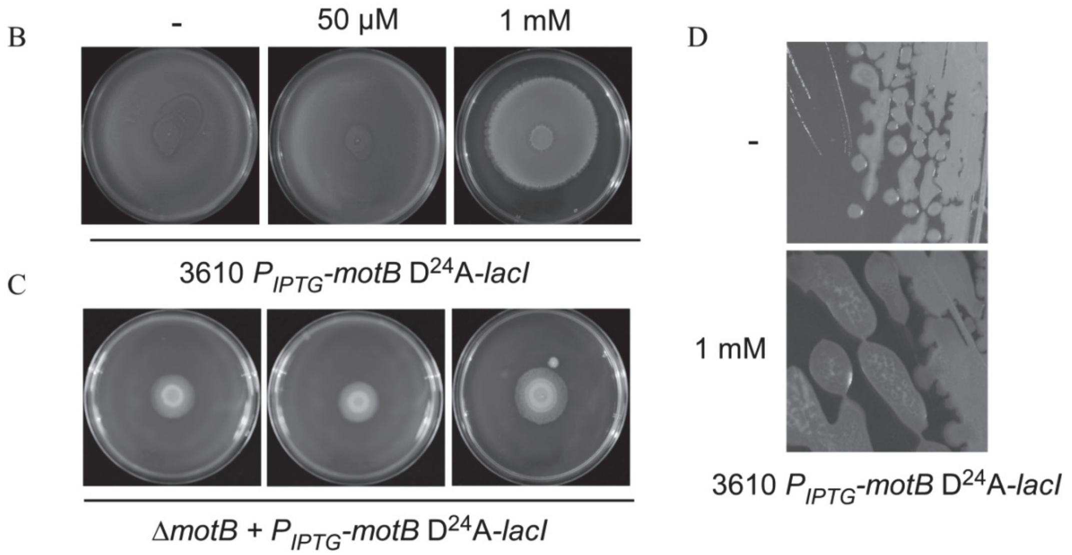

Figure S2 Characterisation of the *motB* D[24]A mutation. (A) Sequence alignment for the MotB proteins from *E. coli* (E.) and *B. subtilis* (B.) The conserved aspartic acid residue is shown in bold and underlined text. **(B)** and **(C)** Photographs of swarm expansion assay plates taken after 6 hours incubation at 37 °C. Strains shown are: **(B)** Δ*motB* + P_{IPTG}-*motB* D[24]A-*lacI* (NRS3867) grown in the absence and presence of 50 µM or 1mM IPTG and **(C)** P_{IPTG}-*motB* D[24]A-*lacI* (NRS3868) grown in the absence and presence of 50 µM or 1mM IPTG. **(D)** Colony morphology of P_{IPTG}-*motB*-D[24]A-*lacI* (NRS3868) grown on LB agar in the absence and presence of 1 mM IPTG.

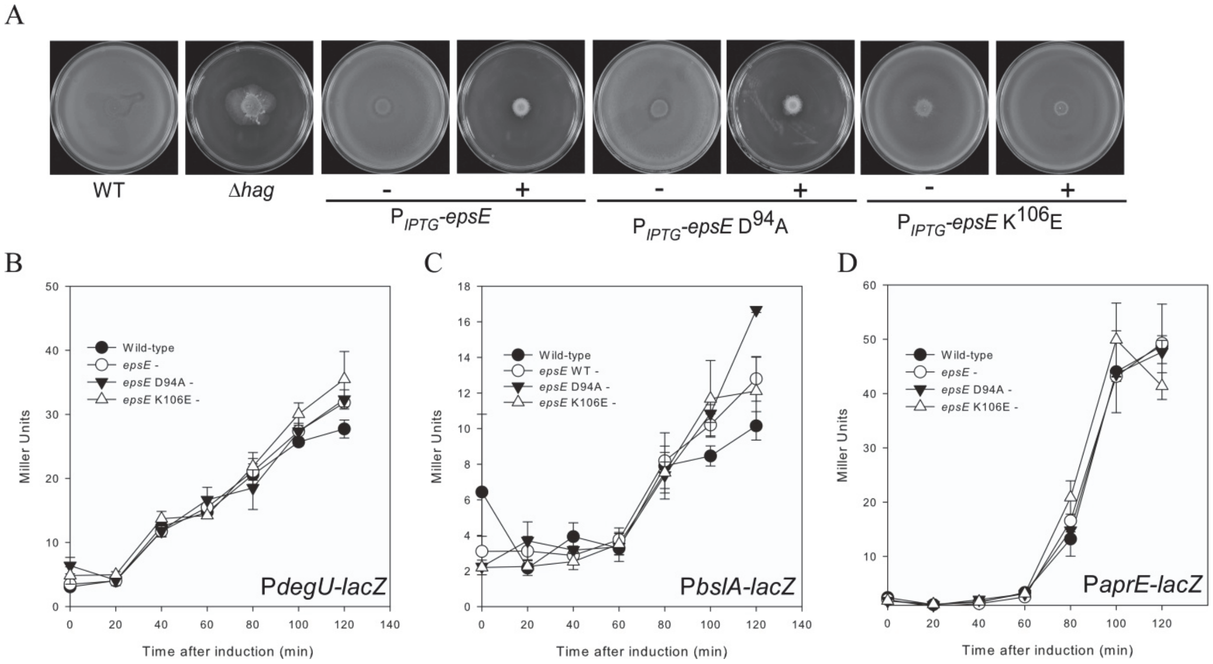

Figure S3 The clutch function of EpsE stops flagellar motility and results in γ-PGA biosynthesis. (A) Swarm expansion assay for the wild-type (WT, NCIB3610), Δ*hag* (DS1677) and P_{IPTG}-*epsE* (NRS4085), P_{IPTG}-*epsE*-D^{94}A (NRS4388) and P_{IPTG}-*epsE*-K^{106}E (NRS4389) in the absence and presence of 1mM IPTG. Photographs of plates were taken after 5 hours incubation at 37 °C. **(B)**, **(C)** and **(D)** β-galactosidase assays of strains carrying the P*degU-lacZ*, P*bslA-lacZ* or P*aprE-lacZ* transcriptional reporter fusions, respectively. Data shown are negative controls grown in the absence of IPTG (see Figure 5). Cells were grown to 0.5 OD_{600}. Strains shown are **(A)** WT (wild-type NRS4351), *epsE* + (P_{IPTG}-*epsE-lacI* (NRS4374)), *epsE* D^{94}A + (P_{IPTG}-*epsE*-D^{94}A-*lacI* (NRS4392)) and *epsE* K^{106}E (P_{IPTG}-*epsE*-K^{106}E-*lacI* (NRS4394)). **(B)** wild-type (NRS2052), P_{IPTG}-*epsE* (NRS4405), P_{IPTG}-*epsE*-D^{94}A (NRS4406) and P_{IPTG}-*epsE*-K^{106}E (NRS4407). **(C)** wild-type (NRS1561), P_{IPTG}-*epsE* (NRS4345), P_{IPTG}-*epsE*-D^{94}A (NRS4393) and P_{IPTG}-*epsE*-K^{106}E (NRS4395). Data shown in parts **(B)**, **(C)** and **(D)** are plotted as the average of at least 3 independent replicates. Error bars represent standard error of the mean.

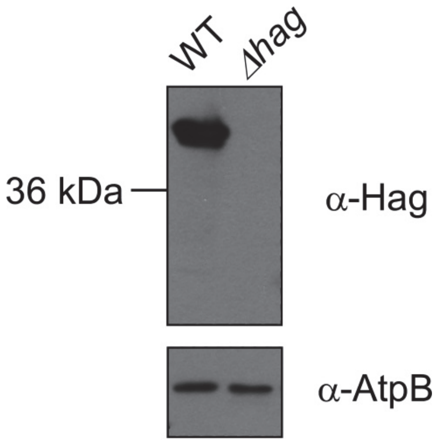

Figure S4 The Hag antibody is highly specific. Western blot of cellular protein fractions collected from WT (wild-type, NCIB3610) and Δ*hag* strain (DS1677) probed with anti-Hag and anti-AtpB as a loading control.

Movie S1 Live cell microscopy of motile *Bacillus subtilis*. Movie of the P*degU-lacZ* strain (NRS451) grown in the absence of antibodies captured 5 minutes after the start of the time-course shown in Fig. 7B. Movies were acquired using phase contrast, 40 X objective with the Zen high speed digital recorder (Zeiss) and are shown at 5 frames per second. Movie files were created with QuickTime Player v10.1. A static image from the data set is shown in Figure 7A.

Movie S2 Tangling of flagella with a Hag antibody. Movie of the P*degU-lacZ* strain (NRS451) captured 5 minutes after the addition of anti-Hag antibody to the culture. Movies were acquired using phase contrast, 40 X objective with the Zen high speed digital recorder (Zeiss) and are shown at 5 frames per second. Movie files were created with QuickTime Player v10.1. A static image from the data set is shown in Figure 7A.

Movie S3 Live cell microscopy of motile *Bacillus subtilis*. Movie of the P*degU-lacZ* strain (NRS451) grown in the absence of antibodies captured 30 minutes after the start of the time-course shown in Fig. 7B. Movies were acquired using phase contrast, 40 X objective with the Zen high speed digital recorder (Zeiss) and are shown at 5 frames per second. Movie files were created with QuickTime Player v10.1.

Movie S4 Live cell microscopy of motile Bacillus subtilis grown with pre-immune sera. Movie of the P*degU-lacZ* strain (NRS451) captured 30 minutes after the addition of pre-immune sera 1 to the culture. Movies were acquired using phase contrast, 40 X objective with the Zen high speed digital recorder (Zeiss) and are shown at 5 frames per second. Movie files were created with QuickTime Player v10.1.

Supplemental Experimental Procedures.

Western Blot.

Cellular proteins were extracted as for whole cell analysis of Hag. 10 µg of cellular proteins were separated by SDS-PAGE prior to transfer onto PVDF membrane (Millipore) by electroblotting. Antibodies raised against Hag (a kind gift from Prof. Kürsad Turgay) were used at 1:40,000, anti-AtpB (Abcam) was used at 1:10,000 and goat anti-rabbit or goat anti-mouse (both from Pierce) HRP-conjugated secondary antibodies were used at 1:5,000.

Plasmid Construction.

Construction of plasmid pNW680. Plasmid pNW1045, used to introduce the *motB* coding region under the control of the IPTG-inducible promoter $P_{hy\text{-}spank}$ at the non-essential *amyE* locus, is a derivative of pDR111 (Britton *et al.*, 2002). The coding region of *motB* (including ribosome binding site) was amplified from *B. subtilis* 3610 chromosomal DNA with primers NSW1312 and NSW1313. The PCR product was digested with HindIII and SphI and cloned into pDR111 cut the same.

Construction of plasmid pNW703. Plasmid pNW703, used to disrupt the *degU* locus, contains an internal region of DNA from the *degU* coding region and a kanamycin resistance cassette. To construct this plasmid the internal region of *degU* was cut from pBL204 (Stanley & Lazazzera, 2005) using BamHI and EcoRI and ligated into pUC19 (Vieira & Messing, 1982) also digested with BamHI and EcoRI creating plasmid pNW701. The kanamycin resistance cassette was cut from plasmid pDG783 (Guerout-Fleury *et al.*, 1995) using SphI and XbaI and ligated into pNW701 also digested with SphI and XbaI creating pNW703.

Construction of pNW1029. Plasmid pNW1029, used to introduce the coding region of motAB under the control of the IPTG-inducible promoter $P_{hy\text{-}spank}$ at the non-essential *amyE* locus, is a derivative of pDR111 (Britton *et al.*, 2002). The coding region of *motAB* (including ribosome binding site) was amplified from *B. subtilis* 3610 chromosomal DNA with primers NSW969 and NSW1313. The PCR product was digested with SalI and SphI and cloned into pDR111 cut the same.

Construction of pNW1044. Plasmid pNW1044, used to introduce the *epsE* coding region under the control of the IPTG-inducible promoter $P_{hy\text{-}spank}$ at the non-essential *amyE* locus, is a derivative of pDR111 (Britton *et al.*, 2002). The coding region of *epsE* (including ribosome binding site) was amplified from *B. subtilis* 3610 chromosomal DNA with primers NSW1602 and NSW1603. The PCR product was digested with SalI and SphI and cloned into pDR111 cut the same.

Construction of pNW1045. Plasmid pNW1045, used to introduce a P*degU-lacZ* transcriptional reporter fusion at the non-essential *thrC* locus, is a derivative of pDG1663 (Guerout-Fleury *et al.*, 1996). The promoter region of *degU* was amplified from *B. subtilis* NCIB3610 chromosomal DNA with primers NSW404 and NSW405. The PCR product was digested with EcoRI and HindIII and cloned into pDG1663 cut the same.

Construction of pNW1047. Plasmid pNW1047, used to introduce *epsE* $D^{94}A$ under control of the IPTG-inducible promoter $P_{hy\text{-}spank}$ at the non-essential *amyE* locus, is a derivative of pNW1044. The D94A mutation was introduced to pNW1044 by site-directed mutagenesis with primers NSW1608 and NSW1609, using PCR amplification conditions calculated according to the Stratagene Quikchange manual using KOD Hot Start DNA polymerase (Novagen). PCR products were then treated with Dpn*I* and transformed into competent *E. coli* MC1061 cells. Plasmid pNW1048 for *epsE* $K^{106}E$ was constructed in an identical manner using primers NSW1610 and NSW1611.

Construction of pNW1060. Plasmid pNW1060, used to introduce *motB* $D^{24}A$ under the control of an IPTG-inducible promoter $P_{hy\text{-}spank}$ at the non-essential *amyE* locus, is a derivative of pNW680. Briefly, pNW680 was cut with HindIII and SphI to excise *motB*, which was ligated into pUC19 cut the same to create plasmid pNW1058. pNW1058 was used as template DNA in a site-directed mutagenesis PCR reaction (Stratagene ™) with primers NSW1011 and NSW1012 to introduce the $D^{24}A$ point mutation and yield plasmid pNW1059. pNW1059 was then cut with HindIII and SphI to excise *motB* $D^{24}A$, which was ligated into pDR111 to create pNW1060.

144 **Table S1 Strains used in this study.**

Strain	Relevant Genotype[a]	Source[b]
**NCIB 3610**	Prototroph	B.G.S.C.
**168**	*trpC2*	B.G.S.C.
**JH642**	*trpC2 pheA1*	(Perego & Hoch, 1988)
**RL2420**	PY79 pIC333	(Kearns *et al.*, 2004)
**DS1677**	3610 Δ*hag*	Kind gift D. Kearns
**NRS1136**	JH642 *degS::cml*	(Verhamme *et al.*, 2007)
**NRS1154**	JH642 *pgdS::pBL141(cml)*	(Stanley & Lazazzera, 2005)
**NRS1314**	3610 *degU::pBL204 (cml)*	(Verhamme *et al.*, 2007)
**NRS1325**	3610 *degU::pBL204 (cml) amyE::*P$_{hy\text{-}spank}$-*degU32-hy-lacI (spc)*	(Verhamme *et al.*, 2007)
**NRS1433**	168 *pgsB::spc*	(Stanley & Lazazzera, 2005)
**NRS1534**	168 *thrC::*P*aprE-lacZ (mls)*	(Verhamme *et al.*, 2007)
**NRS1561**	3610 *thrC::*P*aprE-lacZ (mls)*	(Verhamme *et al.*, 2007)
**NRS2051**	JH642 *thrC::PbslA-lacZ (mls)*	(Verhamme *et al.*, 2009)
**NRS2052**	3610 *thrC::PbslA-lacZ (mls)*	(Verhamme *et al.*, 2009)
**NRS3347**	3610 *pgdS::cml*	NRS1154→NCIB3610
**NRS3348**	3610 Δ*motB* + *pgdS::cml*	NRS1154→NRS3494
**NRS3374**	168 *degU::*pNW703 *(kan)*	pNW703 →168
**NRS3433**	3610 *pgsB::spc*	SPP1 NRS1433 →NCIB3610
**NRS3434**	3610 Δ*motB* + *pgsB::spc*	SPP1 NRS1433 → NRS3494
**NRS3494**	3610 Δ*motB*	pNW654 → NCIB3610
**NRS3514**	168 *amyE::*P$_{hy\text{-}spank}$-*motB-lacI (spc)*	pNW680 → 168
**NRS3744**	3610 Δ*motAB*	pNW1021→ NCIB3610
**NRS3764**	3610 *pgsB::spc* + *degU::kan*	SPP1 NRS3374 →NRS3433
**NRS3769**	168 *amyE::*P$_{hy\text{-}spank}$-*motAB-lacI (spc)*	pNW1029 → 168
**NRS3770**	3610 *pgsB::spc* + *degU::kan amyE::*P$_{hy\text{-}spank}$-*degU32-hy-lacI (cml)*	SPP1 NRS1268 →NRS3764
**NRS3775**	3610 Δ*motB* + P$_{hy\text{-}spank}$-*motB-lacI (spc)*	SPP1 NRS3514 →NRS3494
**NRS3866**	168 P$_{hy\text{-}spank}$-*motB*D^{24}A-*lacI (spc)*	pNW1051→168
**NRS3867**	3610 Δ*motB* + *amyE::*P$_{hy\text{-}spank}$-*motB*D^{24}A-*lacI (spc)*	SPP1 NRS3866 →NRS3494
**NRS3868**	3610 *amyE::* P$_{hy\text{-}spank}$-*motB*D^{24}A-*lacI (spc)*	SPP1 NRS3866 →NCIB3610
**NRS3870**	3610 Δ*motB* + *amyE::*P$_{hy\text{-}spank}$-*motB*D^{24}A-*lacI (spc) thrC::*P*aprE-lacZ (mls)*	SPP1 NRS1534 →NRS3867
**NRS4083**	168 *amyE::*P$_{hy\text{-}spank}$-*epsE-lacI (spc)*	pNW1044 →168
**NRS4084**	168 *thrC::*P*degU-lacZ (mls)*	pNW1045 →168
**NRS4085**	3610 *amyE::*P$_{hy\text{-}spank}$-*epsE-lacI (spc)*	SPP1 NRS4083 →NCIB3610
**NRS4093**	3610 Δ*motAB* + *thrC::*P*aprE-lacZ (mls)*	SPP1 NRS1534 →NRS3744
**NRS4345**	3610 *amyE::*P$_{hy\text{-}spank}$-*epsE-lacI (spc) thrC::*P*aprE-lacZ (mls)*	SPP1 NRS1534 →NRS4085
**NRS4351**	3610 *thrC::*P*degU-lacZ (mls)*	SPP1 NRS4084 →NCIB3610
**NRS4353**	3610 Δ*motB* + *thrC::*P*degU-lacZ (mls)*	SPP1 NRS4084 →NRS3494
**NRS4354**	3610 Δ*motAB thrC::*P*degU-lacZ (mls)*	SPP1 NRS4084 →NRS3744
**NRS4373**	3610 *degU::cml* + *thrC::*P*degU-lacZ (mls)*	SPP1 NRS4084 →NRS1314
**NRS4374**	3610 *amyE::*P$_{hy\text{-}spank}$-*epsE-lacI (spc) thrC::*P*degU-lacZ (mls)*	SPP1 NRS4084 →NRS4085

**Strain**	**Relevant Genotype**[a]	**Source**[b]
**NRS4385**	168 *amyE::*$P_{hy\text{-}spank}$-*epsE*-K^{106}E-*lacI (spc)*	pNW1048 →168
**NRS4386**	168 *amyE::*$P_{hy\text{-}spank}$-*epsE*-D^{94}A-*lacI (spc)*	pNW1047 →168
**NRS4387**	3610 Δ*motAB* + *amyE::*$P_{hy\text{-}spank}$-*motAB-lacI*	SPP1 NRS3469 →NRS3744
**NRS4388**	3610 *amyE::*$P_{hy\text{-}spank}$-*epsE*-D^{94}A-*lacI (spc)*	SPP1 NRS4386 →NCIB3610
**NRS4389**	3610 *amyE::*$P_{hy\text{-}spank}$-*epsE*-K^{106}E-*lacI (spc)*	SPP1 NRS4385 →NCIB3610
**NRS4392**	3610 *amyE::*$P_{hy\text{-}spank}$-*epsE*-D^{94}A-*lacI (spc)* + *thrC::PdegU-lacZ (mls)*	SPP1 NRS4084 →NRS4388
**NRS4393**	3610 *amyE::*$P_{hy\text{-}spank}$-*epsE*-D^{94}A-*lacI (spc)* + *thrC::PaprE-lacZ (mls)*	SPP1 NRS1534 →NRS4388
**NRS4394**	3610 *amyE::*$P_{hy\text{-}spank}$-*epsE*-K106E-*lacI (spc)* + *thrC::PdegU-lacZ (mls)*	SPP1 NRS4084 →NRS4389
**NRS4395**	3610 *amyE::*$P_{hy\text{-}spank}$-*epsE*-K106E-*lacI (spc)* *thrC::PaprE-lacZ (mls)*	SPP1 NRS1534 →NRS4389
**NRS4396**	3610 Δ*motB* + *thrC::*P*degU-lacZ (mls)* *amyE::*$P_{hy\text{-}spank}$-*motB-lacI (spc)*	SPP1 NRS3514 →NRS4353
**NRS4397**	3610 Δ*motB* + *thrC::*P*degU-lacZ (mls)* *amyE::*$P_{hy\text{-}spank}$-*motB-*D^{24}*A-lacI (spc)*	SPP1 NRS3866 →NRS4353
**NRS4398**	3610 Δ*motB* + *degS::cml*	SPP1 NRS1136 →NRS3494
**NRS4399**	3610 *amyE::*$P_{hy\text{-}spank}$-*epsE-lacI (spc)* + *thrC::PaprE-lacZ (mls)*	SPP1 NRS1136 →NRS4345
**NRS4400**	3610 *amyE::*$P_{hy\text{-}spank}$-*epsE*-D^{94}A-*lacI (spc)* + *thrC::PaprE-lacZ (mls)*	SPP1 NRS1136 →NRS4393
**NRS4401**	3610 *amyE::*$P_{hy\text{-}spank}$-*epsE*-K106E-*lacI (spc)* + *thrC::PdegU-lacZ (mls)*	SPP1 NRS1136 →NRS4395
**NRS4405**	3610 *amyE::*$P_{hy\text{-}spank}$-*epsE-lacI (spc)* + *thrC::PbslA-lacZ (mls)*	SPP12051→NRS4345
**NRS4406**	3610 *amyE::*$P_{hy\text{-}spank}$-*epsE*-D^{94}A-*lacI (spc)* + *thrC::PbslA-lacZ (mls)*	SPP12051→NRS4393
**NRS4407**	3610 *amyE::*$P_{hy\text{-}spank}$-*epsE*-K106E-*lacI (spc)* + *thrC::PbslA-lacZ (mls)*	SPP12051→NRS4395

[a] Drug resistance cassettes are indicated as follows: *kan*, kanamycin resistance; *mls*, lincomycin/erythromycin resistance; *cml*, chloramphenicol resistance and *spc*, spectinomycin resistance.

[b] The direction of strain construction is indicated with DNA or phage (SPP1) (→) recipient strain.

B.S.G.C. is the *Bacillus* genetic stock centre.

Table S2 Plasmids used in this study.

**Plasmid**	**Relevant Genotype** [a]	**Source**
**pDR111**	*amyE* integration plasmid *(spc)*	(Britton *et al.*, 2002)
**pMAD**	In-frame markerless deletion plasmid	(Arnaud *et al.*, 2004)
**pDG1663**	*lacZ* reporter fusion plasmid, *thrC* integration	(Guerout-Fleury *et al.*, 1996)
**pUC19**	Cloning plasmid	(Vieira & Messing, 1982)
**pBL204**	pUC19 containing a *cml* resistance cassette	(Stanley & Lazazzera, 2005)
**pNW651**	Region upstream of *motB* in pUC19	This study
**pNW652**	Region downstream of *motB* in pUC19	This study
**pNW653**	Δ*motB* in pUC19	This study
**pNW654**	Δ*motB* in pMAD	This study
**pNW680**	*motB* in pDR111	This study
**pNW701**	pUC19 containing internal region of *degU*	This study
**pNW703**	Kanamycin resistance cassette in an internal region of *degU* coding region	This study
**pNW1019**	Δ*motAB* in pUC19	This study
**pNW1021**	Δ*motAB* in pMAD	This study
**pNW1029**	*motAB* in pDR111	This study
**pNW1044**	*epsE* in pDR111	This study
**pNW1045**	P*degU* in pDG1663	This study
**pNW1047**	*epsE* $D^{94}A$ in pDR111	This study
**pNW1048**	*epsE* $K^{106}E$ in pDR111	This study
**pNW1058**	*motB* in pUC19	This study
**pNW1059**	*motB* $D^{24}A$ in pUC19	This study
**pNW1060**	*motB* $D^{24}A$ in pDR111	This study

[a] Drug resistance cassettes are indicated as follows: *cml*, chloramphenicol resistance; *kan*, kanamycin resistance, and *spc*, spectinomycin resistance.

Table S3 Primers used in this study.

Primer	Target	Sequence [a]	Position [b]
**NSW404**	*degU*	GCATGAATTCGGCCTGATTCCAACTTTAA	-487→-468
**NSW405**	*degU*	GCATAAGCTTCTGATGGTCGTCGATAAT	+26→+39
**NSW874**	*motB*	GTACGGATCCATTGAGGATGTAGATGATGC	-480→-460
**NSW875**	*motB*	ATGCTCTAGAGTACAGCACAATAAACAATGC	+77→+98
**NSW876**	*motB*	GCATTCTAGAGTTGAAGTTCTCATTTTGCCG	+700→+721
**NSW877**	*motB*	GCATGTCGACCATCGCTCCAACATACACC	+1500→+1519
**NSW965**	*motA*	GCTAGGATCCTTGAGGATGAAATGACCGATCTGC	-479→-156
**NSW966**	*motA*	GCTATCAGACGAAGTTTTATCCATAGTTTTCACC	-10→+16
**NSW969**	*motA*	GCTAGTCGACAGACAAGCTAGTAAAAAAGGATTTGG	-66→-9
**NSW1011**	*motB*	TCATGGCTCGTTCCTTACGCCG**C**CATCCTTACTCTTCTCCTG	+49→+91
**NSW1012**	*motB*	CAGGAGAAGAGTAAGGATG**G**CGGCGTAAGGAACGAGCCATGA	+49→+91
**NSW1312**	*motB*	GCATAAGCTTGCAGAACAAGGAGAGGCGCAAAAT	-30→-6
**NSW1313**	*motB*	GCATGCATGCCTATTTTTCATTTGTTTCCGCTGCGC	+880→+904
**NSW1474**	*pgsB*	CTGTTAACCCAGATTATCAAATC	+331→+354
**NSW1475**	*pgsB*	CTGCGCGGCAGTTCATGATGAT	+889→+868
**NSW1602**	*epsE*	AGGAGGTCGACAAAGGAGAAAAGCGTATGAACTCAG	-16→-1
**NSW1603**	*epsE*	CTCCTGCATGCTGGCTGCTATTCATGCTTGACAAG	+820→+843
**NSW1604**	*pgdS*	GGAGACGGCCAAATGGTTC	+370→+388
**NSW1605**	*pgdS*	GCAAGCCGGTCAGAAAAAG	+778→+796
**NSW1608**	*epsE*	CGCACGTCAG**G**CCGGAGATGACCTTTCG	+270→+298
**NSW1609**	*epsE*	CGAAAGGTCATCTCCG**G**CCTGACGTGCG	+270→+298
**NSW1610**	*epsE*	CCGCGCCGTCTGGAA**G**AGCAGGTCGCGTTTTTA	+301→+333
**NSW1611**	*epsE*	TAAAAACGCGACCTTCT**C**TTCCAGACGGCGCGG	+301→+333
**DEN5**	*16S rRNA*	TCACGRCACGAGCTGACGAC	
**DEN7**	*16S rRNA*	ACTCCTACGGGAGGCAGC	

[a] Underlined sequences indicate endonuclease restriction cut sites. Bold sequences represent base pairs mutated by site-directed mutagenesis.

[b] Position of primer is indicated in relation to the translational start site (noted as +1) of the named gene.

Supplemental References

Arnaud, M., Chastanet, A. & Debarbouille, M., (2004) New vector for efficient allelic replacement in naturally nontransformable, low-GC-content, gram-positive bacteria. *Appl Environ Microbiol* **70**: 6887-6891.

Britton, R. A., Eichenberger, P., Gonzalez-Pastor, J. E., Fawcett, P., Monson, R., Losick, R. & Grossman, A. D., (2002) Genome-wide analysis of the stationary-phase sigma factor (Sigma-H) regulon of *Bacillus subtilis*. *Journal of Bacteriology* **184**: 4881-4890.

Guerout-Fleury, A. M., Frandsen, N. & Stragier, P., (1996) Plasmids for ectopic integration in Bacillus subtilis. *Gene* **180**: 57-61.

Guerout-Fleury, A. M., Shazand, K., Frandsen, N. & Stragier, P., (1995) Antibiotic-resistance cassettes for *Bacillus subtilis*. *Gene* **167**: 335-336.

Kearns, D. B., Chu, F., Rudner, R. & Losick, R., (2004) Genes governing swarming in *Bacillus subtilis* and evidence for a phase variation mechanism controlling surface motility. *Mol Microbiol* **52**: 357-369.

Perego, M. & Hoch, J. A., (1988) Sequence analysis and regulation of the *hpr* locus, a regulatory gene for protease production and sporulation in *Bacillus subtilis*. *J Bacteriol* **170**: 2560-2567.

Stanley, N. R. & Lazazzera, B. A., (2005) Defining the genetic differences between wild and domestic strains of *Bacillus subtilis* that affect poly-gamma-dl-glutamic acid production and biofilm formation. *Mol Microbiol* **57**: 1143-1158.

Verhamme, D. T., Kiley, T. B. & Stanley-Wall, N. R., (2007) DegU co-ordinates multicellular behaviour exhibited by *Bacillus subtilis*. *Mol Microbiol* **65**: 554-568.

Verhamme, D. T., Murray, E. J. & Stanley-Wall, N. R., (2009) DegU and Spo0A jointly control transcription of two loci required for complex colony development by *Bacillus subtilis*. *J Bacteriol* **191**: 100-108.

Vieira, J. & Messing, J., (1982) The pUC plasmids, an M13mp7-derived system for insertion mutagenesis and sequencing with synthetic universal primers. *Gene* **19**: 259-268.
